# Absorption Characteristics of a Passive Damper-Augmented Timoshenko Beam Using a Wave-Decomposition Approach

**DOI:** 10.3390/s26133985

**Published:** 2026-06-23

**Authors:** Samikhshak Gupta, Vijaya V. N. Sriram Malladi

**Affiliations:** Vibrations, Intelligent Testing, Active Learning of Structures Group, Mechanical and Aerospace Engineering, Michigan Technological University, 1400 Townsend Dr, Houghton, MI 49931, USA

**Keywords:** Timoshenko beam, finite element analysis, wave decomposition, power absorption coefficient, damper location, sensor layout, laser vibrometry

## Abstract

**Highlights:**

**What are the main findings?**

- Understanding the wave absorption characteristics of a beam augmented with a single- and multiple-point Kelvin–Voigt dashpot(s).
- Normalized by the local flexural wavelength, absorption follows a recurring spatial pattern with minima at half-wavelength intervals.

**What are the implications of the main findings?**

- Wavelength-normalized placement decouples damper location from parameter tuning, yielding a compact design guideline from the dispersion relation alone.
- Multiple jointly tuned dampers suppress local absorption minima to deliver broadband dissipation beyond the reach of a single passive element.

**Abstract:**

Local impedance variations in structural waveguides partially reflect and absorb incident flexural waves, motivating wave-based strategies for passive vibration control. This study develops and experimentally validates a wave-energy framework to quantify and optimize flexural wave absorption by Kelvin–Voigt attachments on a finite Timoshenko beam. A finite element model is validated against Scanning Laser Doppler Vibrometry measurements from a clamped–clamped aluminum beam with a passive damper mounted near one end, with dashpot parameters identified through two independent approaches and the discrepancies attributed to parameter uncertainty. Wave decomposition of the simulated and measured velocity fields yields the power reflection coefficient *ρ*(*ω*) and power absorption coefficient *α*(*ω*) over the 0–15.3 kHz band. The spring stiffness and damping coefficient exhibit frequency-dependent optima and act as complementary, jointly tuned design variables. Expressing dashpot location in wavelength-normalized coordinates reveals a recurring spatial pattern in which absorption minima cluster around half-wavelength multiples, while multiple spanwise positions yield near-peak absorption at any given frequency. This pattern is governed primarily by the flexural wavelength, decoupling placement from parameter tuning, and persists across clamped–clamped, clamped–free, and free–free boundary conditions. Two independently tuned dampers further broaden the effective absorption band by suppressing local minima in *α*(*ω*). These results demonstrate that measurement-driven wave decomposition provides compact, physically grounded guidelines for passive damper placement in beam structures.

## 1. Introduction

The ability to predict and control structural vibration absorption remains a central challenge in engineering, where efficiency, reliability, and mass constraints are paramount. Structural components such as beams, plates, pipes, and stiffeners act as waveguides that transmit vibrational energy in the form of propagating waves [1,2,3], a behavior also observed in full-scale structures such as slender masonry chimneys [4]. Geometric and material discontinuities [5,6,7], including joints [8,9,10,11,12], fasteners, material transitions [13,14], and cracks [15,16], modify wave transmission and redistribute energy along the structure. These discontinuities also complicate the identification of localized stiffness, damping, and mass contributions because boundary compliance and joint behavior are often uncertain and configuration-dependent. As a result, analytical or numerical predictions tend to degrade at mid to high frequencies, where small geometric or material uncertainties become significant [17,18]. Although finite element models can represent the dynamic behavior of these structures in principle, the accuracy of wave propagation predictions is fundamentally limited by the fidelity of the constitutive and boundary inputs, which are rarely known with sufficient precision in practice [17]. These limitations collectively motivate a wave-based measurement approach in which structural responses are decomposed directly into propagating wave components, reducing reliance on model completeness.

Vibration control strategies are broadly classified as active, semi-active, or passive. Active and semi-active systems offer adaptability by synthesizing or adjusting mechanical impedance through actuators, electronics, and feedback control [19,20,21]. Still, the associated power requirements, wiring complexity, and stability considerations limit their use in weight-sensitive and maintenance-constrained applications. Passive approaches are therefore preferred in such contexts and include viscoelastic damping treatments [22,23], tuned mass absorbers [24], dashpot attachments [25], particle dampers [26,27], and locally resonant metastructures [28,29]. However, each of these solutions carries limitations that restrict its applicability. Viscoelastic treatments distributed across entire surfaces add considerable mass, which is impractical in aerospace and transportation applications [17,18]. Tuned mass absorbers and locally resonant metastructures are designed to target a single frequency or narrow band, and their performance degrades rapidly when operating conditions deviate from the design point [24,28]. Dashpot attachments offer a compact and lightweight alternative, but their effectiveness depends critically on the stiffness, damping coefficient, and the spanwise location at which they are attached. Yet, no systematic framework in the literature exists that treats placement as a primary design variable within a wave-based power-absorption context.

Wave-based analysis provides a physically meaningful framework for addressing this gap. By decomposing measured structural responses into incident, reflected, and transmitted wave components, power flow quantities can be extracted that directly characterize how a discontinuity redistributes incoming wave energy. Prior work has applied this framework extensively to characterize joints [9,30], boundaries [31], and structural discontinuities [8] using the power reflection coefficient ρ(ω) and power transmission coefficient τ(ω), and has established important tools for sensor placement and inversion conditioning [32,33]. However, existing work has treated discontinuities exclusively as passive scatterers to quantify how much energy passes through or is reflected at a joint or boundary. The power absorption coefficient α(ω)=1−ρ(ω)−τ(ω), which quantifies the fraction of incident wave power dissipated at an attachment, has not been adopted as a design objective. Additionally, these wave decomposition tools have not been connected to the systematic optimization of passive damper parameters and placement. Unlike modal approaches that prescribe damper parameters at a fixed location to suppress a target mode, the present framework treats location, stiffness, and damping as co-optimized variables with respect to a broadband power-absorption criterion.

The influence of damper location on power absorption, therefore, remains only partially resolved, despite extensive work on Dynamic Vibration Absorbers (DVAs) and passive attachments. Studies on DVAs and tuned passive attachments have primarily focused on identifying optimal stiffness and damping values at a fixed or heuristically chosen location, guided largely by modal considerations at a single target frequency [24,25]. The spanwise variation of the power absorption coefficient over a broad frequency range and the wavelength-normalized patterns governing optimal damper positioning for a given structural configuration have not been systematically examined. This leaves the field without compact, physically grounded design guidelines for the positioning of passive dampers in weight-sensitive structures. To address these limitations, the present work adopts α(ω) as the primary design metric, treats damper location, along with the damping coefficient and stiffness, as explicit design variables, and connects the wave decomposition of both simulated and measured responses to systematic parametric studies of placement and damping. A commercially available air dashpot, Airpot 2KS160 (Airpot Corporation, Norwalk, CT, USA), is mounted on a clamped–clamped aluminum beam, and the measured and simulated responses are processed via wave decomposition to estimate ρ(ω) and α(ω) across a broad frequency range.

Against this background, the present study advances passive vibration control in three principal respects. First, power absorption is established as the primary design objective, evaluated directly through wave decomposed measurements rather than through model tuning alone, with dashpot spring stiffness, damping magnitude, and placement location treated jointly as the governing design parameters. Second, the parametric analysis demonstrates that a distinct optimal value of both the spring stiffness and the damping coefficient maximizes α(ω) at each excitation frequency, with stiffness governing the frequency of peak absorption and damping governing its amplitude; since the optimal pairing migrates with frequency, no single parameter set is universally optimal across the bandwidth, and the two quantities must accordingly be tuned jointly. Third, the analysis establishes that peak absorption recurs at several discrete positions along the span and that, once expressed in coordinates normalized by the local flexural wavelength, these positions collapse onto a periodic spatial pattern; effective placement can therefore be inferred from the dispersion relation alone, independently of the damper tuning, revealing a practical decoupling between the placement decision and the selection of stiffness and damping. Collectively, these contributions yield physically interpretable design maps and compact, wavelength based guidelines for the placement and tuning of passive dampers in mass efficient structural waveguides.

## 2. Materials and Methods

### 2.1. Finite Element and Experimental Model

This section presents the finite element model of the host beam and its experimental validation within a combined numerical and experimental framework developed to investigate how passive damper (dashpot) parameters, namely, stiffness, damping, and location, govern power absorption from a wave propagation perspective. A full glossary of the mathematical symbols used throughout is provided in Appendix A.

#### 2.1.1. FE Model of the Host Beam

To model the host structure and simulate the dashpot–beam interaction, a finite element representation of the aluminum beam was developed and paired with the experimental assembly described in the next subsection. A beam–dashpot assembly was constructed with an aluminum beam as the host structure and a passive damper mounted near the left clamped end of the host structure. The host structure is represented using the Timoshenko beam formulation [34,35,36], with its geometric and material properties provided in Table 1.

A one-dimensional finite element discretization with second-order Timoshenko elements is adopted to capture shear deformation and rotary inertia over the frequency range of interest. The Euler–Bernoulli model was not considered, as its assumptions lead to reduced accuracy at higher frequencies, where shear effects and rotary inertia become non-negligible [37,38,39]. Accordingly, the Timoshenko formulation provides a more suitable basis for the present study. The governing equations describe the coupled flexural displacement w(x,t) and bending rotation ψ(x,t) along the beam axis [40,41].

(1) $$\begin{array}{cc} \frac{\partial }{\partial x}\left[\kappa^{2}GA\left(\frac{\partial w}{\partial x}-\psi \right)\right] & =\rho_{b}A\frac{\partial^{2}w}{\partial t^{2}},\\ \frac{\partial }{\partial x}\left[EI\frac{\partial \psi }{\partial x}\right]+\kappa^{2}GA\left(\frac{\partial w}{\partial x}-\psi \right) & =\rho_{b}I\frac{\partial^{2}\psi }{\partial t^{2}}, \end{array}$$

where $\rho_{b}$ is the material density of the beam, *A* is the cross-sectional area, *I* is the second moment of inertia, *E* is the linear elastic modulus, *G* represents the shear modulus, and κ is the Timoshenko shear coefficient [36,42,43,44]. The host beam is modeled with fixed–fixed boundary conditions, such that the flexural displacement and bending rotation satisfy

$$\begin{array}{cc} w\left(x,t\right)\;=\;\psi \left(x,t\right){|}_{x\;=\;\mathrm{fixed}\;\mathrm{end}} & =0. \end{array}$$

The governing partial differential equations in Equation (1) are discretized using a Galerkin finite element formulation with second-order shape functions [45,46,47,48,49,50]. A mesh of 400 elements is adopted to resolve natural frequencies up to 15.3 kHz, with a convergence study, as discussed in Appendix B, that ensures all modes within this bandwidth are fully captured. This frequency range is of particular significance, as shorter wavelengths at higher frequencies amplify the sensitivity of wave propagation to structural discontinuities and material uncertainties, where purely model-based approaches are known to deteriorate [51,52]. It therefore constitutes a rigorous validation regime for the proposed framework in the context of aerospace and weight-sensitive transportation structures. The assembled global mass and stiffness matrices are then used in MATLAB (v2025a) to compute the beam’s dynamic response. Although spectral elements can provide higher accuracy per degree of freedom, the present finite element discretization is sufficient for the objectives of this study.

The host beam is modeled with Rayleigh damping to represent distributed structural losses. For an *n*-degree-of-freedom system, the equations of motion are

(2) $$\begin{array}{c} M\ddot{x}+C\dot{x}+Kx=0, \end{array}$$

where M, C, and K denote the mass, damping, and stiffness matrices, respectively. The damping matrix is assumed to be mass–stiffness proportional,

(3) $$C=\alpha_{R}M+\beta_{R}K,$$

with proportional-damping coefficients $\alpha_{R}$ and $\beta_{R}$. These parameters are identified via a least-squares fit of experimentally measured modal damping ratios $\zeta_{i}$ to the corresponding natural frequencies $\omega_{i}$ over 0–15.3 kHz [53,54,55], yielding $\alpha_{R}=0.569\;1/s$ and $\beta_{R}=7.846\times 10^{-8}\;s$ discussed in Appendix C.

For the beam with an end-mounted passive damper (PD), the governing equation becomes

(4) $$M\ddot{x}+\left(C+cB_{pd}\right)\dot{x}+\left(K+kB_{pd}\right)x=B_{f}F\left(t\right),$$

where M, C, and $K\in R^{1598\times 1598}$ are the assembled system matrices; *k* and *c* are the PD stiffness and damping; F(t) is the applied external force; $B_{f}$ is the force distribution matrix; and $B_{pd}$ defines the spatial coupling of the PD [56]. The localized dashpot (PD) is modeled as a Kelvin–Voigt element, which introduces non-classical (non-proportional) damping. Since the wave decomposition framework estimates propagating-wave amplitudes directly from the measured response field, it remains applicable in the presence of localized non-proportional damping and captures the net energy dissipated at the damper boundary interface.

#### 2.1.2. Experimental Setup and Validation

**Figure 1:**
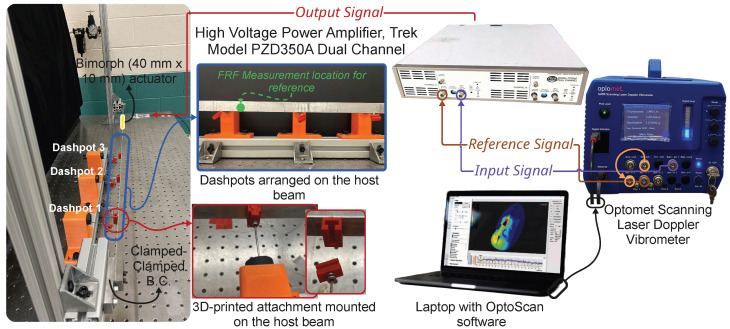
Schematics of the Experimental setup.

Experiments on an aluminum beam were performed to validate the finite element host structure model. A schematic of the setup is shown in Figure 1. The beam was clamped at both ends to reproduce the clamped–clamped boundary conditions assumed in the simulations. Excitation was applied near one end using a piezoelectric parallel bimorph actuator (Piezo Ceramic Bimorph SMBA4510T05M, Steiner & Martins Inc., Davenport, FL, USA) with an active area of 40mm×10mm. Frequency response functions (FRFs) were acquired using a Scanning Laser Doppler Vibrometer (SLDV), (Optomet SWIR Scanning Vibrometer, Darmstadt, Germany) at 119 uniformly spaced spanwise locations; nine representative points were subsequently selected for analysis. A stock air dashpot Airpot 2KS160), with an adjustable damping coefficient ranging from 0–1760 Ns/m, was integrated into the beam assembly via a two-piece interlocking 3D-printed fixture, in which one piece secured to the dashpot rod while the other bonded to the beam surface, enabling precise and repeatable positioning at each prescribed location. Dashpot stiffness and damping were calibrated by minimizing the discrepancy between the simulated and experimental natural frequencies and power absorption coefficients; residual differences, attributed to nearfield effects and non-ideal boundary conditions, introduce uncertainty into the assumed parameter values.

**Figure 2:**
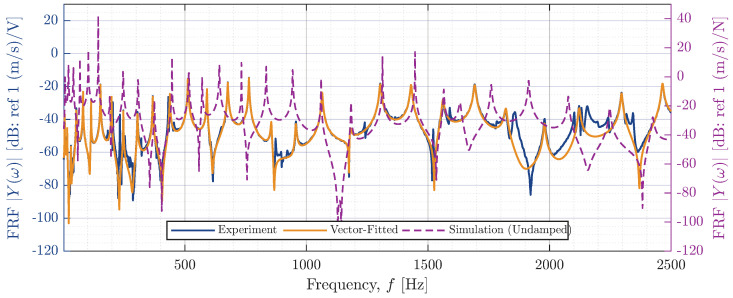
Frequency response of the host structure with 400 elements. The experimental results overlay, validating the FE model. The measurement location is shown in Figure 1.

The experimental FRF was obtained using a Bimorph actuator, for which the input was measured in volts. In contrast, the FE model employed a force based excitation expressed in newtons. As a result, the experimental and simulated FRFs differ in their absolute vertical scaling. This quantitative mismatch is expected, and the comparison instead focuses on the agreement in resonance frequencies and overall spectral trends, which are independent of the input units. As shown in Figure 2, the experimental and simulated FRFs exhibit close alignment, indicating that the FE model captures the dynamic behavior of the host beam with good accuracy. Additional validation metrics, including the relative error between measured and predicted natural frequencies, are presented in Appendix B.

### 2.2. Propagating Wave Decomposition

While FRFs provide a global view of the structural resonances, assessing the localized energy dissipation of the dashpot requires a wave-based perspective. Therefore, the spatial data collected from the nine selected SLDV points is processed using the propagating wave decomposition framework detailed in the following section.

#### 2.2.1. Wave Field Representation and Amplitude Extraction

Structural discontinuities arise at boundaries, applied forces, attachments, or joints between segments. In this work, the discontinuity is realized experimentally as an air dashpot and modeled numerically as a Kelvin–Voigt element representing a non-classical damping device, and the wave reflection and absorption coefficients are determined within the framework established in prior literature [9,30,31].

**Figure 3:**
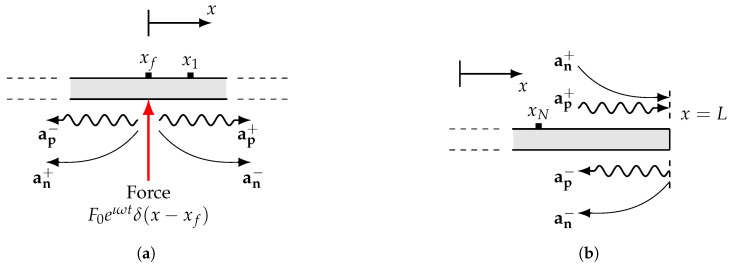
Wave decomposition in excitation and boundary scenarios. (**a**) Propagating and evanescent waves generated by a transverse harmonic point force excitation. (**b**) Reflection of incident forward-going waves into backward-going waves at a boundary.

A transverse harmonic point force $f\left(x,t\right)=F_{0}e^{\iota \omega t}\delta \left(x-x_{f}\right)$, applied at a distance $x_{f}$ from the origin, excites two classes of wave components, propagating waves ($a_{p}^{\pm }$) that carry energy along the beam and evanescent near-field waves ($a_{n}^{\pm }$) that decay rapidly with distance from the source. The superscripts + and − denote waves traveling in the positive and negative *x*-directions, respectively. Both classes are generated on each side of the excitation point, and forward-traveling components that encounter a boundary or discontinuity give rise to reflected waves, as illustrated in Figure 3.

Resolving the wave propagation coefficients requires the wave field to be expressed in terms of its constituent components. For a one-dimensional Timoshenko beam, the transverse displacement w(x,t) is a superposition of four fundamental contributions, two propagating and two evanescent, arising from the governing equations [57,58],

(5) $$w\left(x,t\right)=\left(\underset{\begin{array}{c} \mathrm{forward}\\ \mathrm{propagating} \end{array}}{\underbrace{\hat{A}\;e^{-\iota k_{f}x}}}+\underset{\begin{array}{c} \mathrm{backward}\\ \mathrm{propagating} \end{array}}{\underbrace{\hat{B}\;e^{+\iota k_{f}x}}}+\underset{\begin{array}{c} \mathrm{forward}\\ \mathrm{evanescent} \end{array}}{\underbrace{\hat{C}\;e^{-k_{f}x}}}+\underset{\begin{array}{c} \mathrm{backward}\\ \mathrm{evanescent} \end{array}}{\underbrace{\hat{D}\;e^{+k_{f}x}}}+\underset{\mathrm{higher-order}\;\mathrm{modes}}{\underbrace{\ldots }}\right)\;e^{\iota \omega t},$$

where $k_{f}=k_{f}\left(\omega \right)$ is the complex wavenumber obtained from the Timoshenko dispersion relation and varies quadratically with the excitation frequency ω. The dispersion relation is

(6) $$\frac{EI}{\rho_{b}A}\;{k_{f}}^{4}-\frac{I}{A}\;\left(1+\frac{E}{G\kappa }\right){k_{f}}^{2}\omega^{2}-\omega^{2}+\frac{\rho_{b}I}{GA\kappa }\;\omega^{4}=0,$$

with κ as the Timoshenko shear coefficient, $\rho_{b}$ as the material density, *E* and *G* as the elastic and shear moduli, and *A* and *I* as the cross-sectional area and second moment of area.

The response recorded at a sensor located at $x_{i}$ is the transverse field of Equation (5) evaluated at that location, with measurement noise and nonlinear higher-order effects neglected. Applying the Fast Fourier Transform (FFT) yields the frequency-domain displacement

(7) $$w\left(x_{i},\omega \right)=\hat{A}\left(\omega \right)\;e^{-\iota k_{f}\left(\omega \right)x_{i}}+\hat{B}\left(\omega \right)\;e^{+\iota k_{f}\left(\omega \right)x_{i}}+\hat{C}\left(\omega \right)\;e^{-k_{f}\left(\omega \right)x_{i}}+\hat{D}\left(\omega \right)\;e^{+k_{f}\left(\omega \right)x_{i}},$$

where ι denotes the imaginary unit. Each measurement depends on the four complex wave amplitudes $\hat{A},\hat{B},\hat{C},\hat{D}\in C$, which characterize the relative strengths of the forward and backward propagating and evanescent components. A single sensor cannot uniquely resolve these contributions, so responses from *n* sensors located at $x_{1},x_{2},\ldots ,x_{n}$ are collected into the linear system

(8) $$w\left(\omega \right)=\left\{\begin{array}{c} w(x_{1},\omega )\\ w(x_{2},\omega )\\ \vdots \\ w(x_{n},\omega ) \end{array}\right\}=\left[\begin{array}{cccc} e^{-\iota k_{f}x_{1}} & e^{+\iota k_{f}x_{1}} & e^{-k_{f}x_{1}} & e^{+k_{f}x_{1}}\\ e^{-\iota k_{f}x_{2}} & e^{+\iota k_{f}x_{2}} & e^{-k_{f}x_{2}} & e^{+k_{f}x_{2}}\\ \vdots & \vdots & \vdots & \vdots \\ e^{-\iota k_{f}x_{n}} & e^{+\iota k_{f}x_{n}} & e^{-k_{f}x_{n}} & e^{+k_{f}x_{n}} \end{array}\right]\left\{\begin{array}{c} \hat{A}\left(\omega \right)\\ \hat{B}\left(\omega \right)\\ \hat{C}\left(\omega \right)\\ \hat{D}\left(\omega \right) \end{array}\right\},$$

where w(ω) is the vector of measured responses, which may be displacement, velocity, or acceleration depending on the sensing modality. This overdetermined system is solved in a least-squares sense to estimate the wave amplitudes. The criteria that ensure a stable, far-field estimate are developed next.

#### 2.2.2. Sensor Placement Criteria and Numerical Conditioning

Accurate extraction of the propagating wave amplitudes requires the measured response to be dominated by far-field behavior, achieved by placing the sensors far enough from sources, boundaries, and discontinuities that the evanescent near-field components have decayed to negligible levels. The propagating-only approximation

(9) $$w\left(x_{i},\omega \right)\approx \hat{A}\left(\omega \right)\;e^{-\iota k_{f}\left(\omega \right)x_{i}}+\hat{B}\left(\omega \right)\;e^{+\iota k_{f}\left(\omega \right)x_{i}}\;,$$

holds once the evanescent contributions decay by at least 90% of their magnitude over the propagation distance *l* [59,60], so the sensors must lie outside the near-field region,

(10) $$\left\{\begin{array}{c} x_{1}-x_{f}>l,\\ L-x_{N}>l, \end{array}\right.$$

where the distance *l* satisfies $e^{-k_{f}l}=0.1$. An offset of l=0.10m between the sensors and any excitation point, boundary, or attached dashpot yields a near-field cutoff frequency of 200Hz, above which the far-field assumption holds throughout the analysis frequency range.

To prevent spatial aliasing, the inter-sensor spacing must also satisfy the half-wavelength condition across the bandwidth of interest [9]. With $\lambda_{\min}$ occurring at $f_{\max}=15.3\;\mathrm{kHz}$, the maximum allowable spacing is $\Delta_{\max}=0.015\;m$, while the SLDV spatial resolution imposes a minimum spacing of $\Delta_{\min}=0.011\;m$. These constraints are summarized in Table 2.

**Figure A7:**
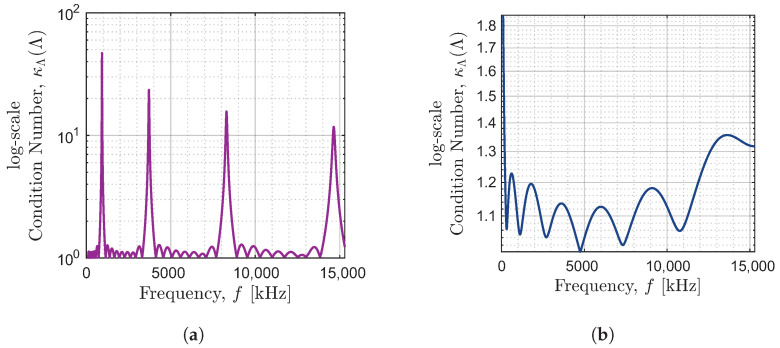
Condition number for the sensor layouts shown in (**a**) uniform spacing and (**b**) non-uniform spacing.

A non-uniform sensor layout is adopted to avoid the large condition numbers $\kappa_{\Lambda }\left(\Lambda \right)$ that degrade the inversion of Equation (8) [32,33]. As compared in Figure A7, a uniform layout produces condition numbers exceeding $10^{2}$ at several frequencies, so small perturbations in the measured response are strongly amplified in the reconstructed amplitudes. The non-uniform arrangement instead keeps $\kappa_{\Lambda }\left(\Lambda \right)$ below 1.8 across the full bandwidth, providing a stable basis for the inversion. The nine adopted sensor locations are listed in Appendix D.

#### 2.2.3. Wave Decomposition by Least-Squares Inversion

Under the far-field approximation of Equation (9), the *n* sensor response reduces to the two propagating unknowns

(11) $$\begin{array}{c} \underset{\begin{array}{c} \mathrm{measured}\;\mathrm{response}\\ w \end{array}}{\underbrace{\left\{\begin{array}{c} w(x_{1})\\ w(x_{2})\\ \vdots \\ w(x_{n}) \end{array}\right\}}}=\underset{\begin{array}{c} \mathrm{design}\;\mathrm{matrix}\\ \Lambda \end{array}}{\underbrace{\left[\begin{array}{cc} e^{-\iota k_{f}x_{1}} & e^{+\iota k_{f}x_{1}}\\ e^{-\iota k_{f}x_{2}} & e^{+\iota k_{f}x_{2}}\\ \vdots & \vdots \\ e^{-\iota k_{f}x_{n}} & e^{+\iota k_{f}x_{n}} \end{array}\right]}}\;\underset{\begin{array}{c} \mathrm{wave}\;\mathrm{amplitudes}\\ x \end{array}}{\underbrace{\left\{\begin{array}{c} \hat{A}\left(\omega \right)\\ \hat{B}\left(\omega \right) \end{array}\right\}}}\;, \end{array}$$

and the wave amplitudes are recovered from the Moore–Penrose pseudo-inverse of the (n×2) design matrix Λ [61],

(12) $$\left\{\begin{array}{c} \hat{A}\left(\omega \right)\\ \hat{B}\left(\omega \right) \end{array}\right\}=\Lambda^{†}\;w,\;\Lambda^{†}={\left(\Lambda^{*}\Lambda \right)}^{-1}\Lambda^{*}.$$

With n=9 sensors and two unknowns, the overdetermined system yields a least-squares estimate whose numerical stability is governed by $\kappa_{\Lambda }\left(\Lambda \right)$ and hence by the non-uniform placement established above. This recovery underlies the power-based coefficients developed for the two damper configurations considered below.

#### 2.2.4. Internally Mounted Dashpot Configuration

**Figure 4:**
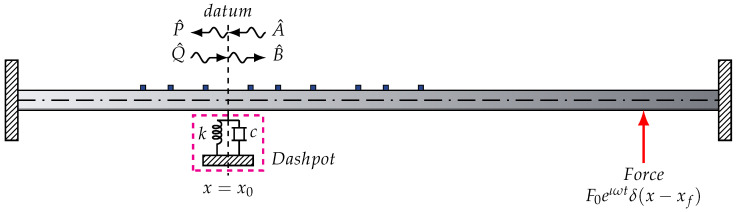
Schematic diagram of a clamped–clamped beam with an internally mounted spring–damper element.

The general configuration is an internally mounted dashpot, which admits both reflection and transmission of the incident wave field, as shown in Figure 4.

The response on each side is decomposed into forward and backward propagating amplitudes, denoted $\hat{A}\left(\omega \right)$ and $\hat{B}\left(\omega \right)$ upstream and $\hat{P}\left(\omega \right)$ and $\hat{Q}\left(\omega \right)$ downstream. With $n_{1}=6$ sensors upstream and $n_{2}=3$ sensors downstream, the design matrices $\Lambda_{1}$ and $\Lambda_{2}$ follow from Equation (11) as

(13) $$\begin{array}{cc} \left\{\begin{array}{c} w(x_{4})\\ \vdots \\ w(x_{9}) \end{array}\right\}\; & =\underset{\begin{array}{c} \mathrm{design}\;\mathrm{matrix}\;1\\ \Lambda_{1} \end{array}}{\underbrace{\left[\begin{array}{cc} e^{-\iota k_{f}x_{4}} & e^{+\iota k_{f}x_{4}}\\ \vdots & \vdots \\ e^{-\iota k_{f}x_{9}} & e^{+\iota k_{f}x_{9}} \end{array}\right]}}\;\left\{\begin{array}{c} \hat{A}\left(\omega \right)\\ \hat{B}\left(\omega \right) \end{array}\right\}\\ \left\{\begin{array}{c} w(x_{1})\\ w(x_{2})\\ w(x_{3}) \end{array}\right\}\; & =\underset{\begin{array}{c} \mathrm{design}\;\mathrm{matrix}\;2\\ \Lambda_{2} \end{array}}{\underbrace{\left[\begin{array}{cc} e^{-\iota k_{f}x_{1}} & e^{+\iota k_{f}x_{1}}\\ e^{-\iota k_{f}x_{2}} & e^{+\iota k_{f}x_{2}}\\ e^{-\iota k_{f}x_{3}} & e^{+\iota k_{f}x_{3}} \end{array}\right]}}\;\left\{\begin{array}{c} \hat{P}\left(\omega \right)\\ \hat{Q}\left(\omega \right) \end{array}\right\}, \end{array}$$

and the amplitudes are recovered through the corresponding pseudo-inverses as

(14) $$\left\{\begin{array}{c} \hat{A}\left(\omega \right)\\ \hat{B}\left(\omega \right) \end{array}\right\}=\Lambda_{1}^{†}\left\{\begin{array}{c} w(x_{4})\\ \vdots \\ w(x_{9}) \end{array}\right\}\;\left\{\begin{array}{c} \hat{P}\left(\omega \right)\\ \hat{Q}\left(\omega \right) \end{array}\right\}=\Lambda_{2}^{†}\left\{\begin{array}{c} w(x_{1})\\ w(x_{2})\\ w(x_{3}) \end{array}\right\}.$$

At the discontinuity, the reflected and transmitted amplitudes relate to the incident amplitudes through the reflection and transmission coefficients r(ω) and t(ω),

(15) $$\left\{\begin{array}{c} \hat{B}\left(\omega \right)\\ \hat{P}\left(\omega \right) \end{array}\right\}=\left[\begin{array}{cc} \hat{A}\left(\omega \right) & \hat{Q}\left(\omega \right)\\ \hat{Q}\left(\omega \right) & \hat{A}\left(\omega \right) \end{array}\right]\left\{\begin{array}{c} r\\ t \end{array}\right\},$$

where $\hat{A}\left(\omega \right)$ and $\hat{Q}\left(\omega \right)$ are the incident waves from the upstream and downstream sides, while $\hat{B}\left(\omega \right)$ and $\hat{P}\left(\omega \right)$ are the reflected and transmitted waves. Inverting Equation (15) gives

(16) $$\left\{\begin{array}{c} r(\omega )\\ t(\omega ) \end{array}\right\}={\left[\begin{array}{cc} \hat{A}\left(\omega \right) & \hat{Q}\left(\omega \right)\\ \hat{Q}\left(\omega \right) & \hat{A}\left(\omega \right) \end{array}\right]}^{-1}\left\{\begin{array}{c} \hat{B}\left(\omega \right)\\ \hat{P}\left(\omega \right) \end{array}\right\}.$$

The power coefficients follow from the squared magnitudes of these wave coefficients. The power reflection and transmission coefficients are $\rho \left(\omega \right)={\left|r\left(\omega \right)\right|}^{2}$ and $\tau \left(\omega \right)={\left|t\left(\omega \right)\right|}^{2}$, while the power absorption coefficient captures the remaining fraction of incident power,

(17) $$\begin{array}{c} \rho \left(\omega \right)={\left|r\left(\omega \right)\right|}^{2},\;\tau \left(\omega \right)={\left|t\left(\omega \right)\right|}^{2},\;\alpha \left(\omega \right)=1-\rho \left(\omega \right)-\tau \left(\omega \right). \end{array}$$

#### 2.2.5. End-Mounted Dashpot Configuration and Baseline Response

**Figure 5:**
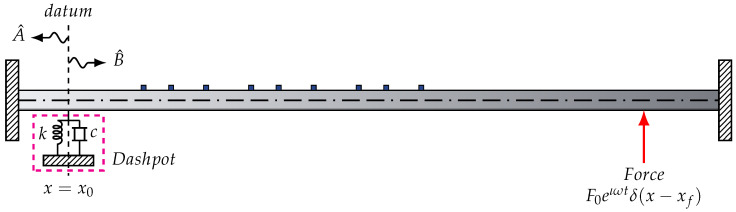
Schematic diagram of a clamped–clamped beam with a spring damper element at one end.

For the end-mounted configuration in Figure 5, the termination is purely reflective (t=0), which simplifies Equation (17) to

(18) $$\rho ={\left|r\right|}^{2}\;,\;\alpha =1-{\left|r\right|}^{2}\;\mathrm{or}\;\alpha =1-\rho \;,$$

with the reflection coefficient defined as

(19) $$r=\frac{\hat{A}\left(\omega \right)}{\hat{B}\left(\omega \right)}\;.$$

The measured quantity $w(x_{i})$ is the velocity at sensor $x_{i}$, treated as a transfer function, velocity per unit force in the FE model, and velocity per unit voltage in the experiment. This unit mismatch introduces a common scaling factor across all entries of the response vector that is inherited equally by $\hat{A}\left(\omega \right)$ and $\hat{B}\left(\omega \right)$. Because the power coefficients are formed from amplitude ratios, the factor cancels exactly, rendering α(ω) invariant to the excitation modality, so experimental variability is attributable mainly to phase-sensitive reconstruction rather than magnitude scaling. As the phase of *r* is sensitive to wavenumber estimation errors and small positioning uncertainties, the analysis relies on the power quantities, which remain considerably more robust and physically interpretable even though they are not entirely immune to measurement uncertainty.

Although the present study addresses the end-mounted case, the framework of Equation (17) extends naturally to internal placements. For an internally mounted dashpot in a finite beam, the energy transmitted past the discontinuity reflects at the downstream boundary and returns upstream, where the far-field upstream array captures it through $\hat{B}\left(\omega \right)$. The quantity $\alpha =1-{\left|r\right|}^{2}$ then represents the net fraction of incident power removed by the dashpot beam system rather than a purely local absorption, since isolating local absorption would require an independent downstream measurement of *t* via Equation (17), which is not feasible here because reliable wave decomposition is limited to frequencies above 200Hz. With only a single impedance discontinuity present, the upstream reconstruction remains a self-consistent measure of net energy removal.

**Figure 6:**
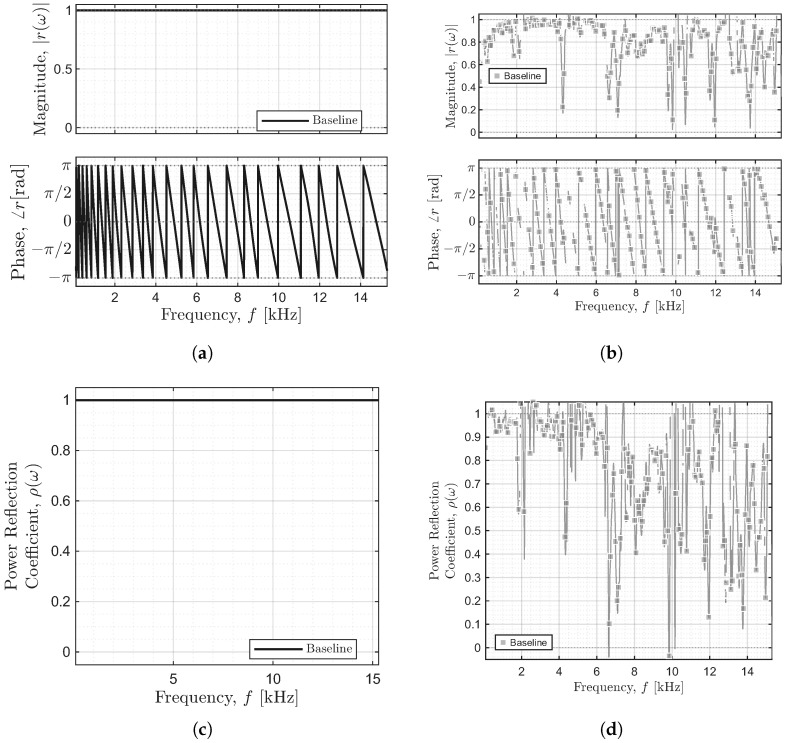

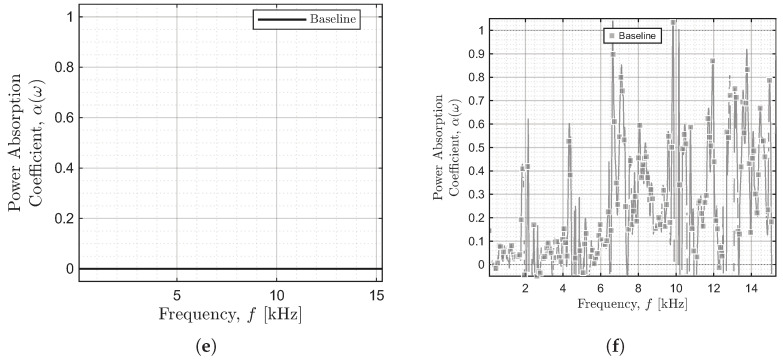
Wave amplitude characteristics of the host beam for the undamped FE model; the dotted line marks the valid limits for the wave amplitudes, between 0 and 1. (**a**) Reflection coefficient magnitude and phase for the host beam from the finite element (FE) model. (**b**) Reflection coefficient magnitude and phase for the host beam from the experimental measurements. (**c**) Power reflection coefficient magnitude for the host beam from the finite element (FE) model. (**d**) Power reflection coefficient magnitude for the host beam from the experimental measurements. (**e**) Power absorption coefficient magnitude for the host beam from the finite element (FE) model. (**f**) Power absorption coefficient magnitude for the host beam from the experimental measurements.

The wave propagation coefficients r(ω), ρ(ω), and α(ω) for the host beam are presented in Figure 6, with the undamped model serving as the baseline reference against which the effects of material damping and dashpot attachments are systematically evaluated in Appendix C.3. In the absence of a dashpot, the boundary conditions alone govern the wave propagation behavior, with |ρ(ω)|≈1 across the frequency range of interest. This baseline provides a clean reference against which the effect of the passive damper (PD) is unambiguously quantified, and the following section introduces the dashpot and examines how it redistributes incident wave energy into reflection and absorption, with particular emphasis on damping, placement, and their combined influence on broadband vibration control.

## 3. Results

This section presents the computed wave coefficients for the host beam with an end-mounted dashpot and examines how the PD alters the resulting wave propagation behavior. The analysis highlights how the added element modifies the energy reflection and absorption characteristics, providing insight into passive vibration control strategies. The following subsections investigate the influence of damping magnitude, dashpot location, and related parameters on the reflection and absorption of structural waves.

### 3.1. Study 1: Influence of a Passive Damper on the Absorption Characteristics of the Host Beam

This study evaluates how adding a dashpot modifies the beam response across frequency using the power reflection coefficient ρ(ω) and the power absorption coefficient α(ω).

**Figure 7:**
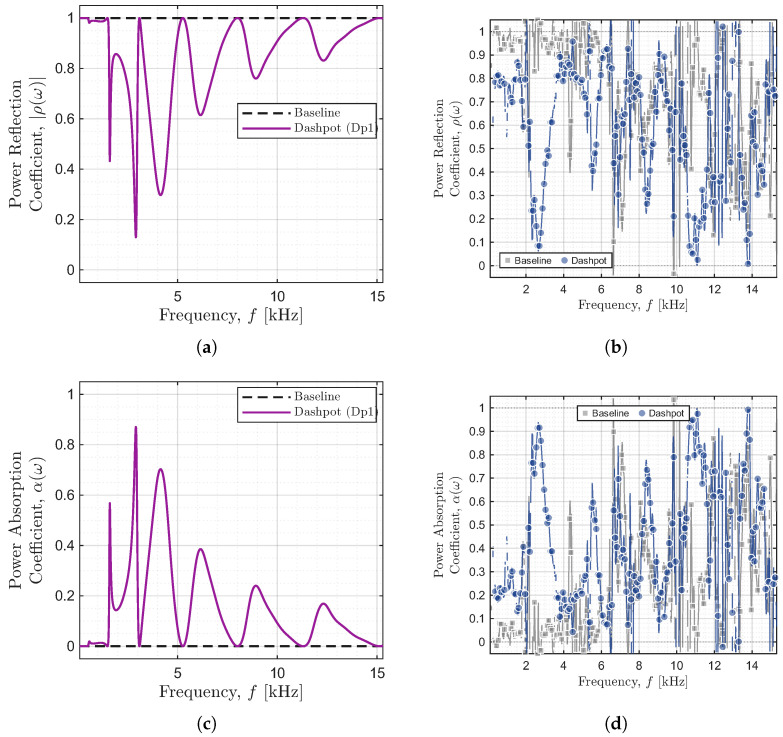
Simulated and experimental power reflection and power absorption coefficients illustrating the wave propagation characteristics of a beam with an end-mounted dashpot. (**a**) Simulated magnitude of the power reflection coefficient for the host beam with an end-mounted dashpot. (**b**) Experimental magnitude of the power reflection coefficient for the host beam with an end-mounted dashpot. (**c**) Simulated magnitude of the power absorption coefficient for the host beam with an end-mounted dashpot. (**d**) Experimental magnitude of the power absorption coefficient for the host beam with an end-mounted dashpot.

A dashpot was installed at x=0.1100m from the beam end. In the simulation, the attachment was modeled with k=1379.01kN/m and c=9.8149Ns/m, identified through calibration against the experimental measurements as described in Appendix F. Residual parameter uncertainty, quantified in Appendix G, contributes to the discrepancy in absolute absorption levels between Figure 7b,c. Despite this offset, both datasets show a repeatable increase in α(ω) over the frequency bands relative to the baseline, indicating that the passive spring–damper markedly modifies the host beam’s absorption behavior, consistent with Figure 7.

**Figure A9:**
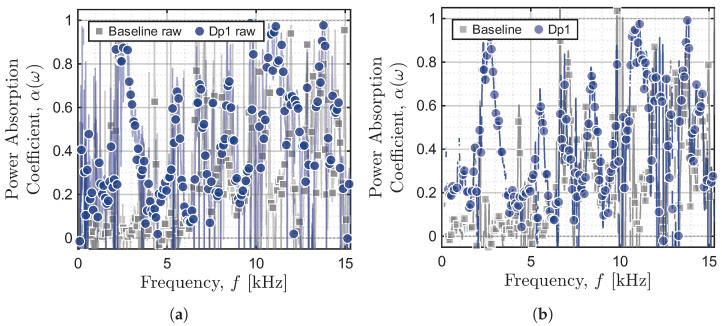
Power absorption coefficient α(ω) for the dashpot (Dp1, blue circles) and baseline (grey squares) configurations. (**a**) Raw decomposition output. (**b**) After coherence masking and Savitzky–Golay smoothing.

The raw experimental power absorption coefficient α(ω) without any filtering occasionally exhibits values outside the physically admissible range [0,1], as shown in Figure A9a. These excursions reflect reconstruction artifacts rather than physical gain, arising from error propagation in the wave amplitude estimates $\hat{A}\left(\omega \right)$ and $\hat{B}\left(\omega \right)$. The design matrix Λ, constructed from $e^{\pm \iota k_{f}x_{i}}$, is sensitive to small uncertainties in the estimated wavenumber $k_{f}$ and sensor positions $x_{i}$, while experimental FRFs may additionally contain phase and amplitude perturbations due to noise, imperfect coherence, and deviations from ideal boundary conditions. The absence of such behavior in the simulations confirms that these excursions are attributable to measurement and reconstruction limitations rather than the underlying physical model.

Several measures are adopted in this study to mitigate these effects. A non-uniform sensor layout is employed to maintain κ(Λ)<1.8 across the full bandwidth, and the overdetermined system (n=9 sensors, two unknowns) is solved via the Moore–Penrose pseudoinverse to suppress the influence of individual sensor noise, discussed more in Appendix D. The analysis focuses on power-based quantities ρ(ω) and α(ω), which are considerably more robust to measurement uncertainty than the phase-sensitive reflection coefficient r(ω). Coherence-based filtering and anti-resonance masking, described in Appendix E, are applied to exclude frequency regions of low signal quality from the decomposition results. Vector fitting [62] was additionally applied to the baseline FRFs to smooth broadband noise while preserving the underlying spectral features, yielding a cleaner estimate of $k_{f}$ used to populate Λ.

Further reduction in non-physical values could be achieved through additional measures in future experimental campaigns. Terminating the beam ends with anechoic materials or sandboxes [8,9] would suppress reflected energy returning to the measurement region, thereby isolating the local energy dissipation at the discontinuity more cleanly and reducing spurious reflected wave contributions in $\hat{B}\left(\omega \right)$. Iterative wavenumber refinement, in which reconstructed wave amplitudes from an initial decomposition are used to update the dispersion-based estimate of $k_{f}$ in successive passes, could reduce the systematic bias that propagates into $\hat{A}\left(\omega \right)$ and $\hat{B}\left(\omega \right)$ from an imperfect initial dispersion relation. Physical-bound enforcement via constrained least-squares, imposing 0≤α≤1 directly, offers a pragmatic safeguard, though it carries the risk of masking genuine reconstruction uncertainty and is therefore best reserved for cases where the source of non-physical behavior has been independently diagnosed.

### 3.2. Study 2: Influence of Passive Damper Stiffness and Damping on the Absorption Characteristics of the Host Beam

It is hypothesized that, for a given dashpot location, the stiffness and damping of the passive spring damper significantly influence the host beam’s absorption characteristics. In particular, varying the spring stiffness *k* and the damping coefficient *c* is expected to shift both the magnitude and the frequency at which the power absorption coefficient α(ω) is maximized, as well as modify the associated power reflection coefficient ρ(ω). The frequency-dependent quantities α(ω) are therefore adopted as the primary metrics for testing this hypothesis.

The equation of motion of the beam with an end-mounted dashpot is expressed in Equation (4). The influence of the dashpot parameters *k* and *c* is investigated by maximizing the frequency-dependent power absorption coefficient, α(ω)

(20) $$\left(k_{\mathrm{opt}},\;c_{\mathrm{opt}}\right)=\max_{k,\;c\;\in \;R^{+}}\;\alpha \left(\omega \right),$$

where α(ω) represents the power absorption coefficient for a given frequency.

As described in Section 2.1.2, an Airpot 2KS160 air dashpot was employed in the experimental configuration, with a manufacturer specified damping coefficient in the range 0–1760Ns/m. For the numerical parameter sweep, this range was extended marginally to c∈[0,2000]Ns/m to provide a conservative search margin, and the stiffness was varied over k∈[0,2500]kN/m. Systematic sweeps over both parameters were conducted at each frequency to identify the combination yielding maximum power absorption as given in Table 3, and the resulting optimal values of *k* and *c* were subsequently analyzed in detail.

**Figure 8:**
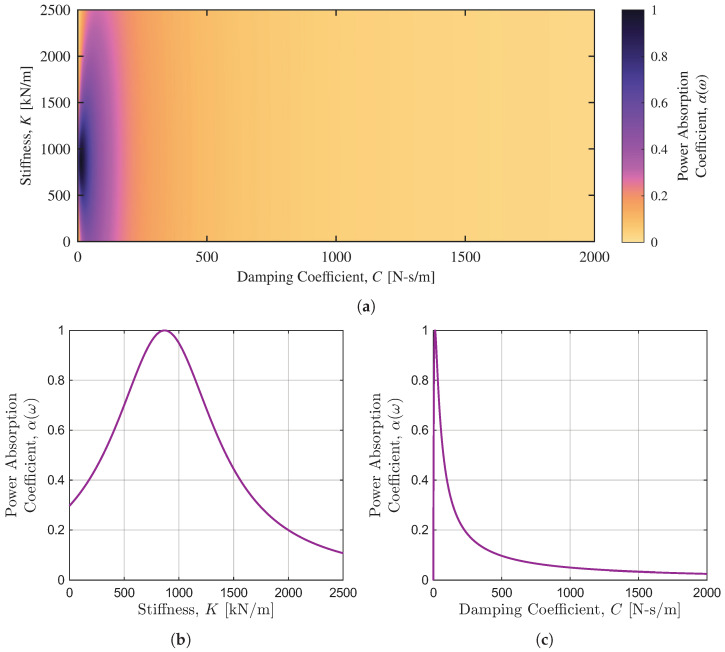
Combined influence of stiffness and damping on the power absorption coefficient at 3500Hz. Panels (**a**–**c**) correspond to the variation over (k,c), *k* only, and *c* only, respectively. (**a**) Power absorption coefficient as a function of stiffness *k* and damping *c* at 3500Hz, highlighting the region of maximum absorption. (**b**) Power absorption coefficient as a function of stiffness *k* at 3500Hz, showing the limited influence of *k*. (**c**) Power absorption coefficient as a function of damping *c* at 3500Hz, showing a clear maximum (optimal damping).

**Figure 9:**
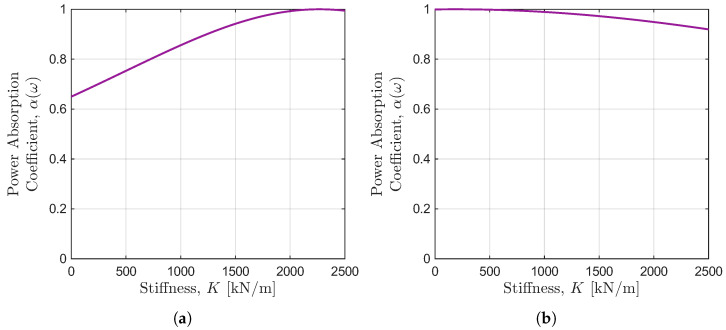

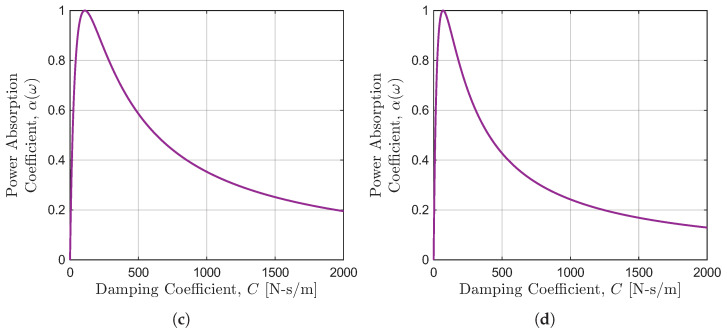
Power absorption coefficient α(ω) versus stiffness *k* for (**a**) f=2270.5Hz and (**b**) f=8993.7Hz, and versus damping coefficient *c* for (**c**) f=2270.5Hz and (**d**) f=8993.7Hz. A distinct absorption optimum is observed for both parameters at each frequency.

Inspection of the parameter sweep results reveals that both the stiffness *k* and the damping coefficient *c* exert a significant and distinct influence on the power absorption coefficient α(ω). As shown in Figure 8 and Figure 9, a clear optimum is identified for both parameters at each excitation frequency; stiffness primarily governs the frequency location of peak absorption, while the damping coefficient controls its amplitude. These observations confirm that both parameters play a role in determining the absorption behavior of the dashpot and must therefore be considered jointly.

**Figure 10:**
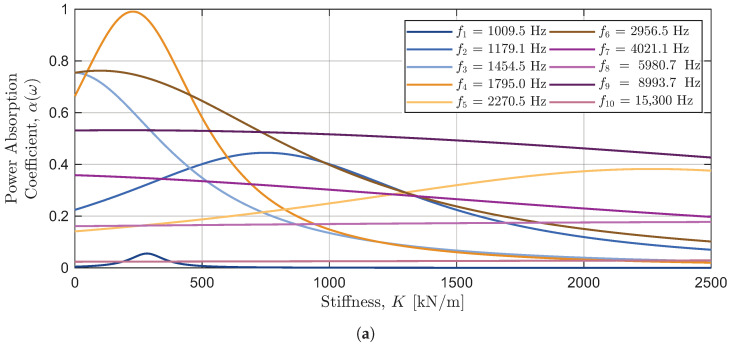

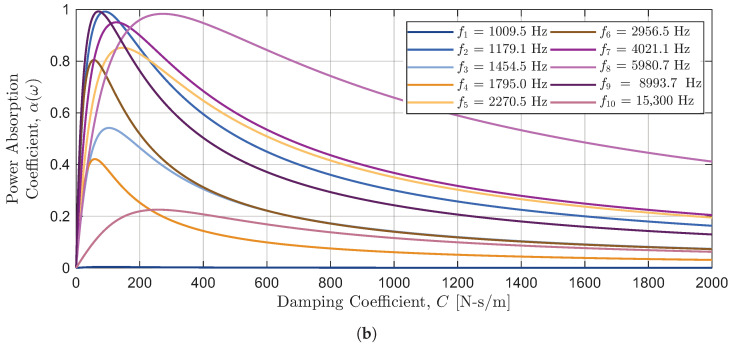
Power absorption coefficient α versus (**a**) stiffness *k* and (**b**) and damping coefficient *c*, for ten excitation frequencies. The absorption peak shifts with frequency for both parameters, demonstrating the strong frequency dependence of the optimal values.

The frequency dependence of the optimal parameter values is examined further across ten discrete excitation frequencies in Figure 10. For both *k* and *c*, the absorption peak shifts to a different parameter value at each frequency, confirming that neither parameter can be assigned a single value that maximizes absorption across the full bandwidth.

**Figure 11:**
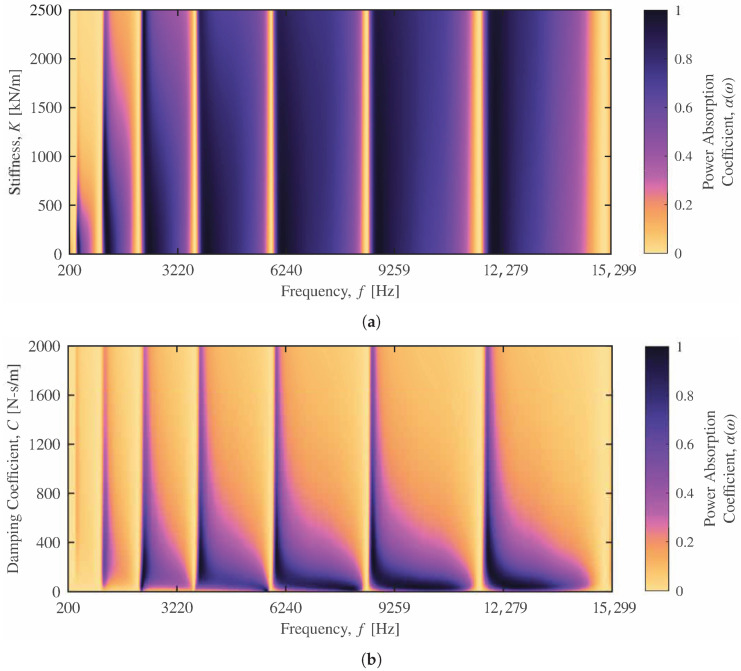
Contour maps of the power absorption coefficient α as a function of (**a**) stiffness *k* (**b**) and damping coefficient *c*, versus excitation frequency, showing regions of strong and weak sensitivity to each parameter.

The contour maps in Figure 11 illustrate how *k* and *c* jointly govern the power absorption characteristics across the full frequency range. Both maps reveal that the locus of maximum absorption traces a distinct path through the parameter-frequency space, consistent with the frequency dependence of the optimal values identified in Figure 10. It is also evident that certain frequency bands exhibit low absorption regardless of the parameter value, indicating that the capacity of the dashpot to dissipate wave energy is inherently limited at those frequencies for the present beam configuration. Within the sensitive bands, however, both *k* and *c* offer meaningful scope for tuning the absorption response.

### 3.3. Study 3: Influence of the Location of Passive Damper on the Absorption Characteristics of the Host Beam

It is hypothesized that, for fixed dashpot stiffness and damping, the spanwise location of the passive spring damper significantly influences the host beam’s absorption characteristics. The results in Section 3.1 and Figure 7 already show that introducing a spring damper modifies both ρ(ω) and α(ω), indicating a measurable impact on the beam’s absorption behavior.

The influence of dashpot location along the beam span on the power absorption coefficient is examined in this section. From Section 3.2, it is established that α(ω) depends on both the stiffness *k* and the damping coefficient *c* of the passive damper at a fixed excitation frequency.

**Figure 12:**
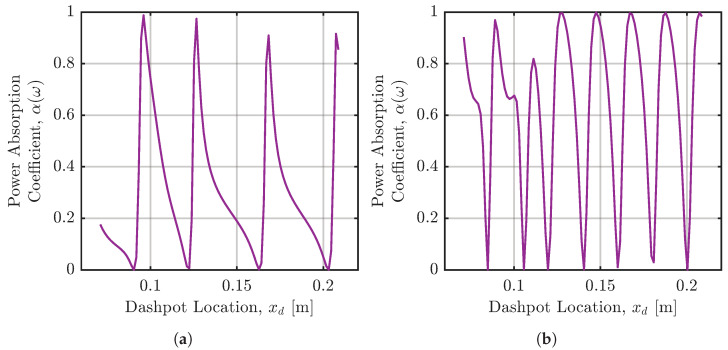
Power absorption coefficient, α, as a function of dashpot location for two excitation frequencies: (**a**) 2270.5Hz and (**b**) 8993.7Hz. The results show that α(ω) is strongly dependent on location and that multiple dashpot positions can yield peak absorption for the same damping coefficient, indicating non-unique optimal placement.

To isolate the effect of position, both the stiffness *k* and the damping coefficient *c* are set to the values that maximize α(ω) at the frequency of interest, and the passive damper is translated from 0.07m to 0.21m, measured from the left clamped end, under the maintained assumption of no transmission. The stiffness and damping values used in this sweep are held constant throughout and summarized in Table 4. As shown in Figure 12, α(ω) varies with dashpot location even when both *k* and *c* are held at their optimal values for a given frequency, demonstrating that position constitutes an independent degree of freedom in the absorption behavior. Furthermore, Figure 12 reveals that, at the tuned frequencies of 2270.5Hz and 8993.7Hz, multiple dashpot locations yield peak absorption for the same combination of *k* and *c*, indicating that the optimal placement is not unique. Taken together, these results confirm that the spanwise location of the passive damper has a pronounced and independent influence on the host beam’s absorption characteristics, complementary to the roles of stiffness and damping established in Section 3.2.

### 3.4. Study 4: Influence of Passive Damper Location Normalized by Wavelength on the Absorption Characteristics of the Host Beam

It is hypothesized that, for fixed spring damper properties, the host beam’s absorption characteristics are governed primarily by the damper location expressed in a form normalized by wavelength. The results in Figure 12 show that the power absorption coefficient exhibits a repeatable spatial pattern as the dashpot is translated along the span. At the higher excitation frequency, the number of absorption peaks increases from 4 to 7. At 2270.5Hz, the wavelength is $\lambda_{1}=0.08\;m$; halving this to $\lambda_{2}=0.04\;m$ (so that $\lambda_{2}=\lambda_{1}/2$) and applying the dispersion relation yields a corresponding frequency of 8993.7Hz, consistent with Table 4. Thus, when the wavelength is halved, the spatial peak pattern approximately doubles, and the principal peaks of the power absorption coefficient scale harmonically with wavelength.

**Figure 13:**
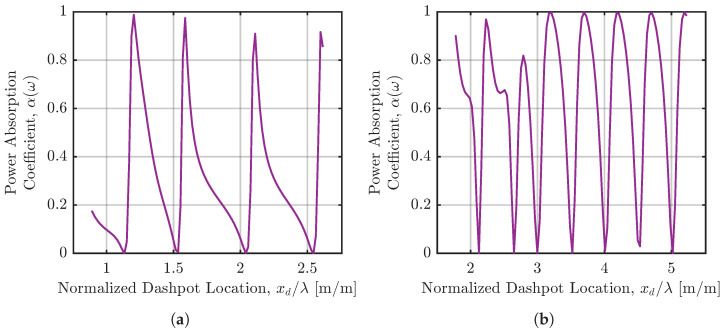
Power absorption coefficient, α, as a function of dashpot location normalized by wavelength for two excitation frequencies: (**a**) 2270.5Hz and (**b**) 8993.7Hz.

**Figure 14:**
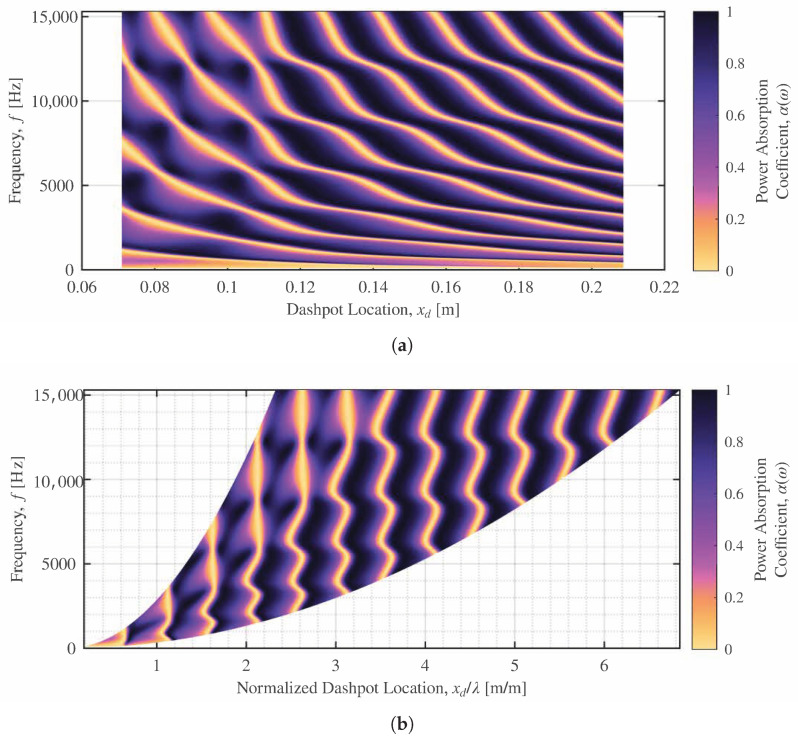
Contour maps of the power absorption coefficient α(ω) as a function of (**a**) absolute dashpot location $x_{d}$ and (**b**) wavelength-normalized dashpot location $x_{d}/\lambda$, both plotted against excitation frequency.

Taken together, these observations demonstrate that the absorption pattern is governed by the dashpot location relative to the local flexural wavelength rather than by its absolute spanwise position, as illustrated in Figure 13. The power absorption coefficient α(ω), plotted in Figure 14 as a contour map in absolute spatial coordinates, is shown as a function of dashpot location $x_{d}$ and excitation frequency *f*. A comparison of the two contour maps reveals this directly; the absolute location plot in Figure 14a shows diagonal absorption ridges that shift continuously with frequency, whereas the wavelength-normalized plot in Figure 14b collapses these ridges into vertical bands that repeat at equal intervals in $x_{d}/\lambda$. The most physically informative feature of the normalized map is the regular recurrence of near-zero absorption at predictable positions. The minima of α(ω) are observed to recur at approximately integer multiples of the half-wavelength, expressed as

(21) $$x_{d}\approx \frac{n\lambda }{2},\;n=1,\;2,\;3,\;\ldots$$

At these locations, the dashpot is unable to extract wave energy effectively regardless of frequency, suggesting a systematic spatial condition under which the attachment becomes ineffective. Between consecutive minima, the absorption coefficient rises to high values, and the pattern repeats with each additional wavelength of distance from the boundary. This spatial periodicity in $x_{d}/\lambda$ confirms that the flexural wavelength is the natural length scale governing dashpot placement, and that the absorption response is determined by how many wavelengths separate the dashpot from the clamped end rather than by the physical distance itself.

The wavelength-normalized placement rule points toward a broader design principle for passive wave absorption in structural waveguides. The underlying physical reasoning is that the effectiveness of the dashpot is determined by the local wave kinematics at the attachment point, which are periodic in $x_{d}/\lambda$.

### 3.5. Study 5: Influence of Superposed Dual Passive Damper Responses on the Absorption Characteristics of the Host Beam

The superposed response of two passive dampers attached to the host beam at distinct optimized locations is hypothesized to yield absorption characteristics that differ fundamentally from those of each damper acting in isolation and cannot be recovered by a linear superposition of the individual responses.

**Figure 15:**
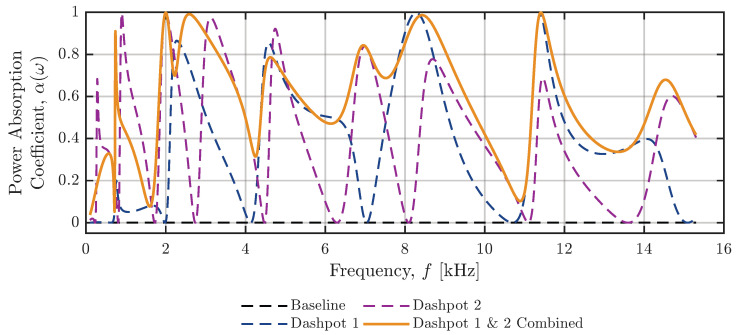
Power absorption coefficient α(ω) for the superposed dual dashpot configuration and each individual dashpot. Dashpot 1 and Dashpot 2 are optimized independently at 2270.5Hz and 8993.7Hz, respectively, with parameters given in Table 5.

Two target excitation frequencies are selected from the parametric study in Section 3.2, and the stiffness, damping coefficient, and spanwise location of each passive damper are optimized independently so that each configuration achieves near-maximum power absorption at its assigned frequency. The resulting parameter sets are summarized in Table 5. Both passive dampers are then attached simultaneously to the host beam, and the combined power absorption coefficient is compared against the individual responses in Figure 15.

The superposed power absorption coefficient differs qualitatively from the individual responses; whereas the single-damper α(ω) curves exhibit several pronounced local minima across the frequency range, these minima are substantially attenuated and effectively eliminated in the superposed α(ω) power absorption coefficient. This behavior indicates a non-trivial interaction between the two passive dampers, providing further support for the hypothesis formulated in this study.

## 4. Discussion

**Figure 16:**
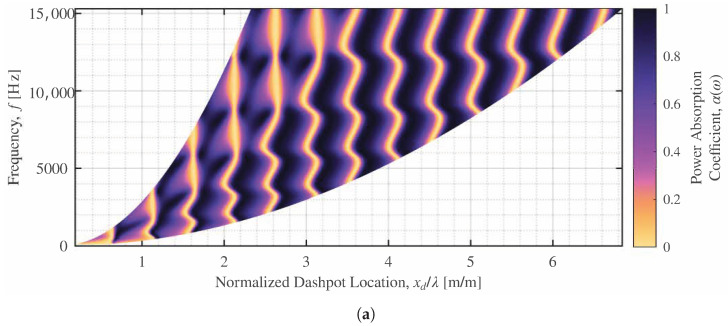

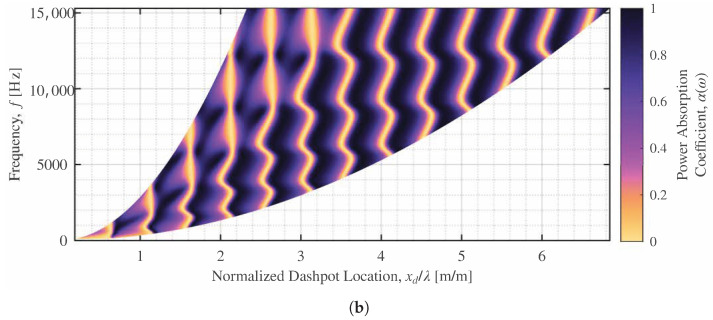
Power absorption coefficient α(ω) as a function of wavelength-normalized dashpot location $x_{d}/\lambda$ and excitation frequency for (**a**) clamped–free and (**b**) free–free boundary conditions. The half-wavelength band spacing and the zero-absorption condition at $x_{d}\approx n\lambda /2$ observed for the clamped–clamped case in Figure 14b are reproduced in both configurations.

The wavelength-normalized placement maps are extended to clamped–free and free–free boundary conditions to examine whether the half-wavelength periodicity identified for the clamped–clamped beam in Figure 14b is configuration specific. As shown in Figure 16, the recurrence of near-zero absorption at $x_{d}\approx n\lambda /2$ is preserved across both the additional boundary conditions, indicating that the placement rule reflects the local wave kinematics at the dashpot attachment rather than the global modal structure imposed by the end conditions.

**Figure 17:**
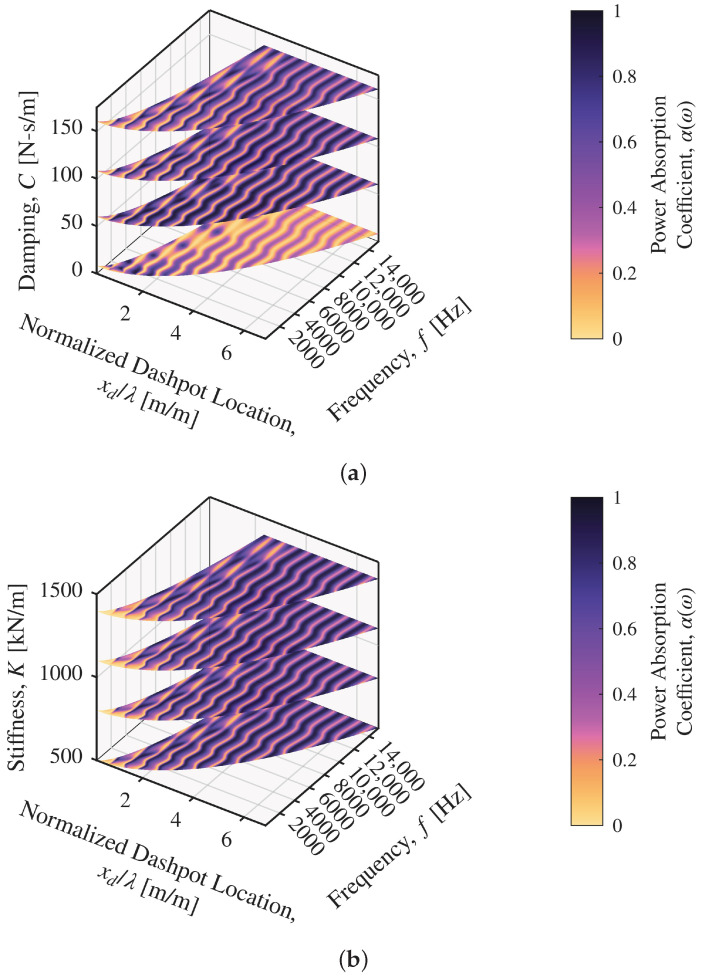
Contour maps of α(ω) as a function of $x_{d}/\lambda$, excitation frequency, and (**a**) damping coefficient *c* and (**b**) stiffness *k*.

This comparison encompasses three boundary conditions, and a more extensive study spanning a wider range of end conditions would be necessary to fully establish the universality of the half-wavelength placement rule. The contour maps in Figure 17 further demonstrate that the band pattern in $x_{d}/\lambda$ is preserved across a wide range of both *c* and *k*, with parameter variations affecting only the amplitude of the absorption peaks and not their spatial locations. This decoupling confirms that dashpot placement can be determined from the wavelength alone, independently of the precise damper tuning.

## 5. Conclusions

This study examined how a passive dashpot mounted near one end of a finite Timoshenko beam modifies flexural wave propagation, quantified through the power reflection and absorption coefficients ρ(ω) and α(ω) recovered by wave decomposition of a nine sensor velocity field over the 0–15.3 kHz band. The finite element model and the Scanning Laser Doppler Vibrometry measurements were in close agreement, confirming that the model captures the dynamic behavior of the host beam, and a single attached element raised α(ω) over distinct frequency bands relative to the undamped baseline. Both the spring stiffness and the damping coefficient were found to possess a well-defined, frequency-dependent optimum and to exert significant, complementary influences that must be tuned jointly, with stiffness primarily setting the frequency of peak absorption and damping its amplitude, so that no single parameter set is optimal across the full bandwidth.

The placement of the damper proved equally influential. Expressed in coordinates normalized by the local flexural wavelength, α(ω) follows a periodic spatial pattern in which near-zero absorption is concentrated around integer multiples of the half-wavelength and rises to high values in between. Because this pattern depends only on the local dispersion relation and not on the damper tuning, placement and parameter selection are effectively decoupled, and the rule persists across clamped–clamped, clamped–free, and free–free boundaries, reflecting local wave kinematics rather than the global modal structure. Two independently tuned dampers further suppress the local minima of α(ω) observed for the single damper case, producing an effectively broadband response that cannot be reproduced by any linear superposition of the individual spectra.

Several limitations of the present study point toward avenues for future investigation. The physical Airpot dashpot has an adjustable damping coefficient ranging from 0 to 1760 Ns/m that is difficult to maintain precisely, and the idealized Kelvin–Voigt representation omits amplitude-dependent and frequency-dependent stiffness effects, causing the model to yield a more regular absorption pattern than observed experimentally. In the frequency domain, the Kelvin–Voigt element contributes a complex impedance of the form k+jωc, which provides a tractable linear characterization and integrates naturally into the finite element assembly, but this representation is inherently restricted to linear constitutive behavior and cannot account for the amplitude-dependent response that physical dashpots exhibit under large-amplitude excitation. Extending the formulation to incorporate nonlinear dashpot elements would therefore produce the measured absorption characteristics and broaden the applicability of the model. The present study adopts three structural damping models for the host beam; strain-rate damping and additional viscoelastic representations could be incorporated to extend the framework to a wider range of structural materials and configurations. Employing a more readily tunable absorber, such as an eddy current damper, would allow closer experimental matching and more direct validation of the placement rules. At the experimental level, terminating the beam ends with anechoic materials or sandboxes would suppress spurious reflected energy returning to the measurement region, reducing the noise in the decomposed wave amplitudes and improving the reliability of the extracted absorption coefficients, particularly at frequencies near structural antiresonances. Data-driven identification methods such as Dynamic Mode Decomposition (DMD) and Sparse Identification of Nonlinear Dynamics (SINDy) offer a complementary route to capturing the nonlinear dynamic effects introduced by the dashpot without requiring an explicit parametric constitutive model, and their integration with the wave decomposition framework has not been explored in the present study but represents a promising direction for future research. Future work should also examine the placement principle across a broader range of structural geometries and materials. Together, the wave based metrics, wavelength-normalized placement rules, and superposed dampers constitute a compact and physically interpretable framework for mass efficient, broadband passive vibration control in structural waveguides.

## Figures and Tables

**Table 1:** Geometric and material properties of the host beam structure.

	Geometric Properties	Material Properties				
	L×W×H (mm × mm × mm)	*E* (GPa)	$\rho_{b}$ ($kg/m^{3}$)	ν	*G* (GPa)	κ
Host Beam	1113×19.05×1.58	68.7	2713.1	0.33	25.6	0.851

**Table 2:** Sensor placement constraints.

	Constraint Condition	Result
Far-Field Assumption	$e^{-k_{f}\;l}=0.1,\;\forall \;\omega \in \left[0.2,\;15.3\right]\;k\mathrm{Hz}$	l=0.10m
Spacing Based on Wavelength	$\Delta_{\max}<\frac{\lambda_{\min}}{2}$	$\Delta_{\max}=0.015\;m$
Experimental Spacing	$\Delta_{\min}>\mathrm{SLDV}\;\mathrm{spatial}\;\mathrm{resolution}$	$\Delta_{\min}=0.011\;m$

**Table 3:** Frequencies and corresponding optimal damping coefficients and stiffness values used in the parametric study.

Frequency	Damping Coefficient, c	Stiffness, k
2270.5 Hz	108.05 Ns/m	2267.6 kN/m
8993.7 Hz	69.03 Ns/m	186.61 kN/m

**Table 4:** Frequencies and corresponding optimal passive damper parameters, wavelengths, and number of absorption peaks from the parametric study.

Frequency	Damping Coefficient, c	Stiffness, k	Wavelength, λ	Number of Peaks
2270.5 Hz	108.05 Ns/m	2267.6 kN/m	0.08 m	4
8993.7 Hz	69.03 Ns/m	186.61 kN/m	0.04 m	7

**Table 5:** Individually optimized passive damper parameters used in the dual dashpot superposition study.

	Frequency	Damping Coefficient, *c*	Stiffness, *k*	Location, $x_{d}$
1	2270.5 Hz	108.05 Ns/m	2267.6 kN/m	0.0946 m
2	8993.7 Hz	69.03 Ns/m	186.61 kN/m	0.1252 m

**Figure A1:**
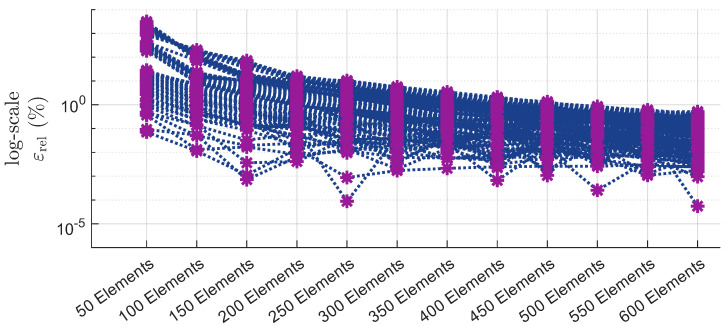
Convergence of the computed natural frequencies with respect to the 1000-element reference model for FE meshes ranging from 50 to 600 elements.

**Figure A2:**
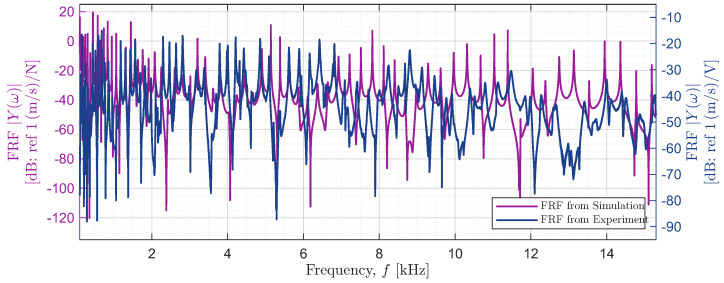
Comparison of the 400-element FE model FRF with experimental measurements, validating the FE model.

**Figure A3:**
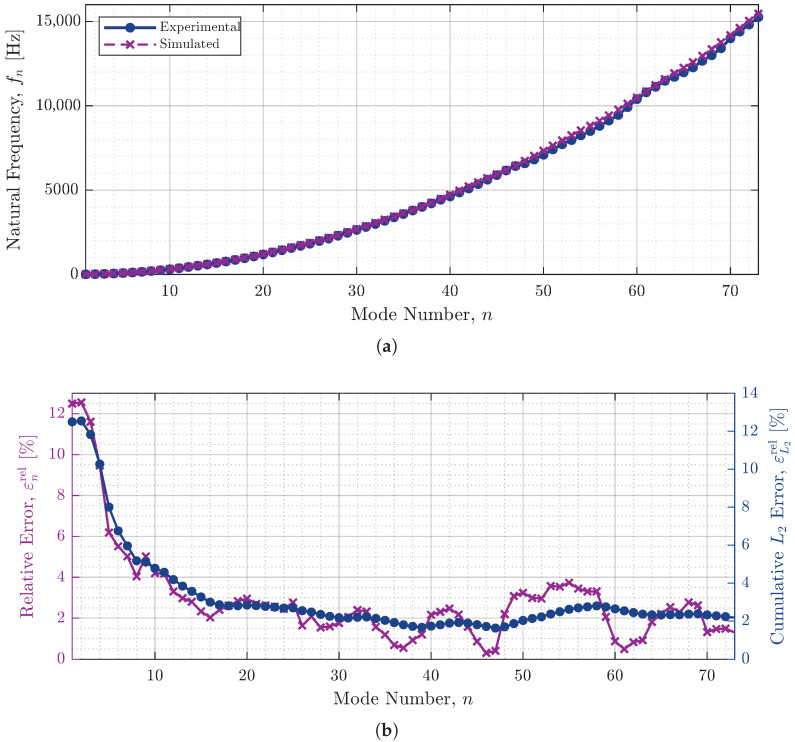
Comparison between experimental and simulated natural frequencies of the host beam. (**a**) Comparison of the measured and FE-predicted natural frequencies. (**b**) Corresponding percentage relative and $L_{2}$-norm errors.

**Figure A4:**
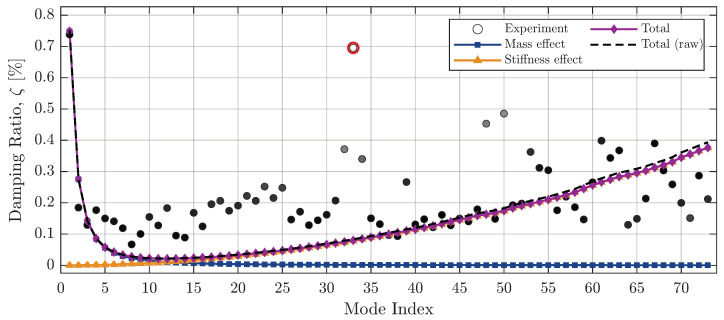
Damping ratio as a function of mode index for the baseline configuration, comparing the experimentally measured damping ratio (black circles) against the simulated proportional damping contributions from mass effects (blue squares), stiffness effects (golden triangles), the combined total (magenta diamonds), and the total computed from raw data; the red circle marks an outlier in the experimental damping ratio.

**Figure A5:**
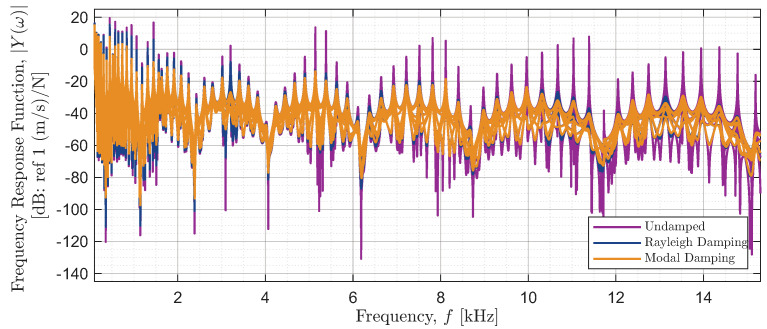
Frequency response functions of the FE model for the undamped, proportionally damped, and modal damped configurations.

**Figure A6:**
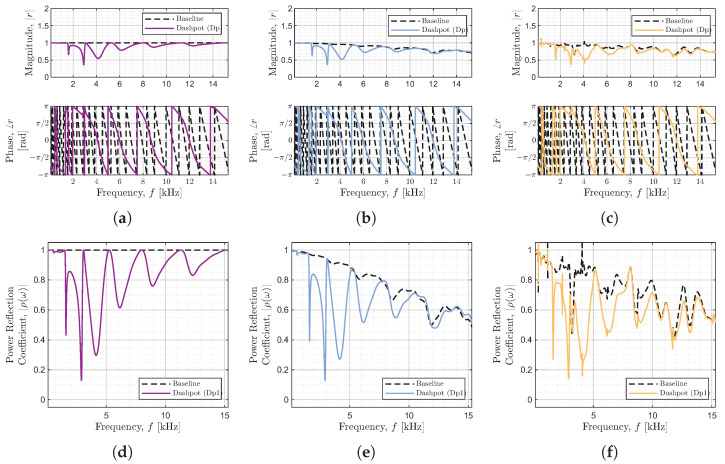

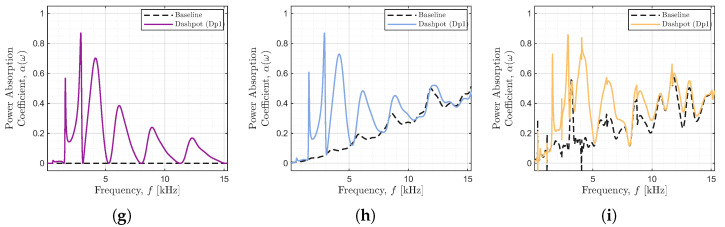
Wave amplitude characteristics of the host beam showing the reflection coefficient, power reflection coefficient, and power absorption coefficient for the undamped, proportionally damped, and modal damped FE models arranged from left to right. (**a**) Reflection coefficient of the undamped model. (**b**) Reflection coefficient for the proportionally damped model. (**c**) Reflection coefficient for the modal damped model. (**d**) Power reflection coefficient of the undamped model. (**e**) Power reflection coefficient for the proportionally damped model. (**f**) Power reflection coefficient for the modal damped model. (**g**) Power absorption coefficient of the undamped model. (**h**) Power absorption coefficient for the proportionally damped model. (**i**) Power absorption coefficient for the modal damped model.

**Figure A8:**
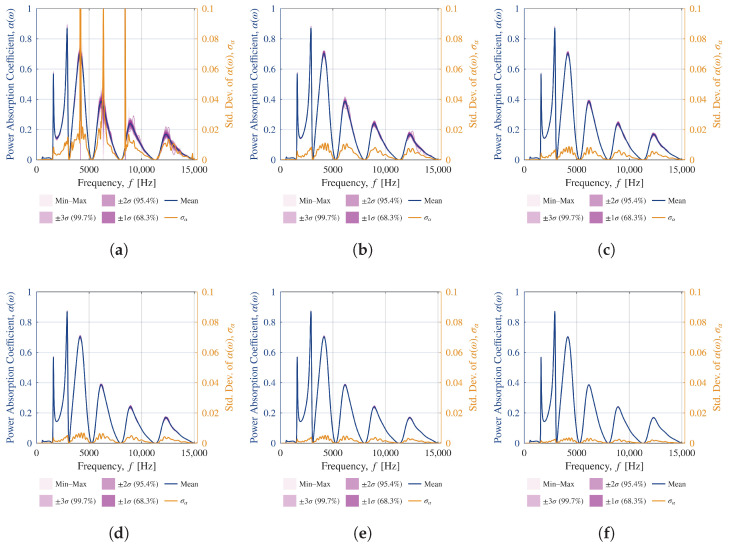
Power absorption coefficient α(ω) for sensor subsets of size n=3–8 from a nine-sensor array. Shaded bands indicate ±1σ–3σ confidence intervals and min–max envelope across all 9n subsets; golden curve (right axis) shows $\sigma_{\alpha }\left(\omega \right)$. (**a**) Sensor count n=3, yielding 93=84 evaluated subsets. (**b**) Sensor count n=4, yielding 94=126 evaluated subsets. (**c**) Sensor count n=5, yielding 95=126 evaluated subsets. (**d**) Sensor count n=6, yielding 96=84 evaluated subsets. (**e**) Sensor count n=7, yielding 97=36 evaluated subsets. (**f**) Sensor count n=8, yielding 98=9 evaluated subsets.

**Figure A10:**
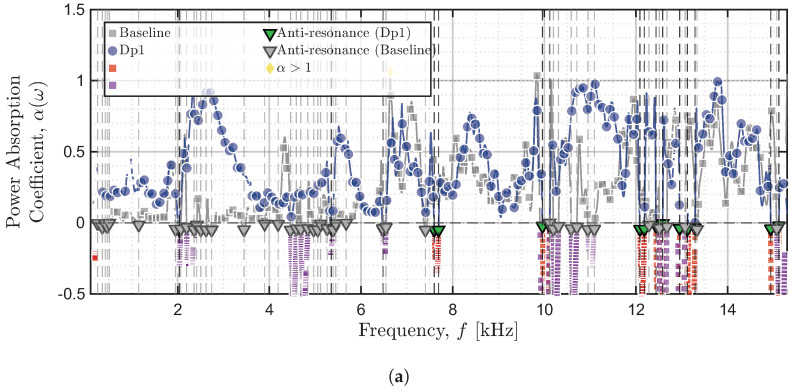

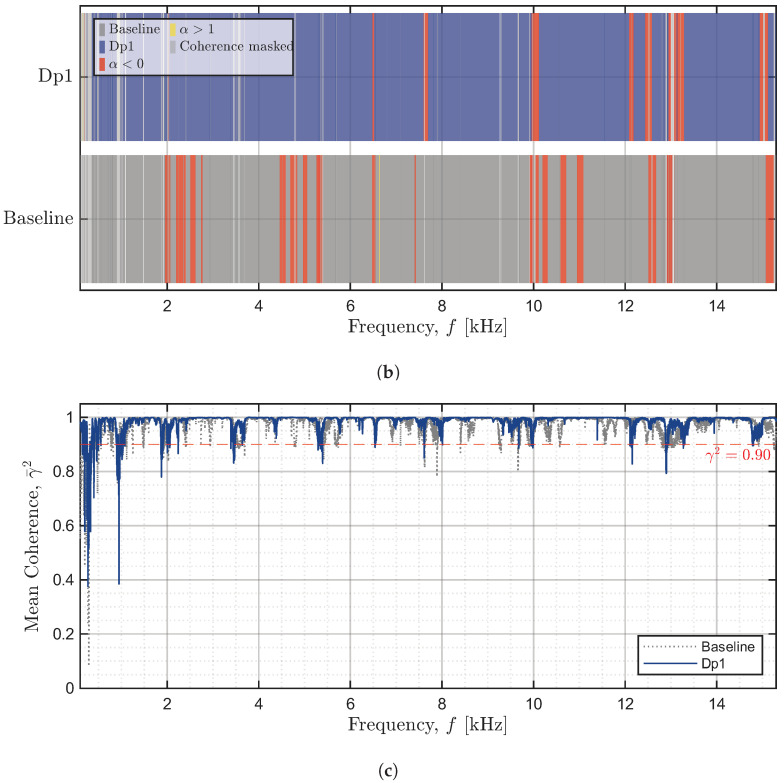
Validity assessment of the experimental power absorption coefficient. (**a**) Smoothed α(ω) after coherence masking for the dashpot (Dp1, blue circles) and baseline (grey squares); golden diamonds mark α>1 and triangles mark antiresonances. (**b**) Frequency-resolved validity map: navy and grey denote valid frequencies, dark red α<0, and light grey coherence masked frequencies. (**c**) Mean coherence spectra ${\bar{\gamma }}^{2}\left(f\right)$; the dashed red line is the exclusion threshold ${\bar{\gamma }}^{2}=0.90$.

**Figure A11:**
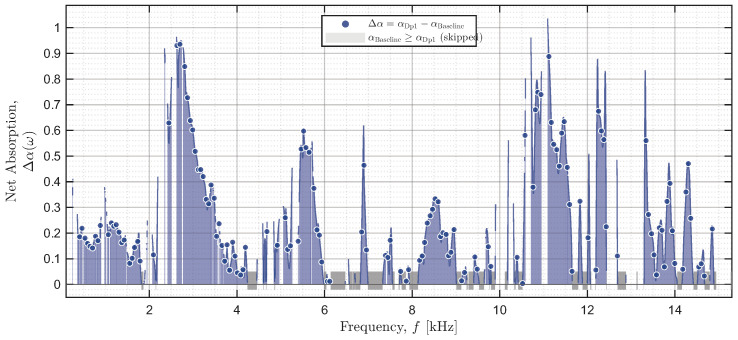
Net power absorption coefficient $\Delta \alpha \left(\omega \right)=\alpha_{\mathrm{Dp}1}\left(\omega \right)-\alpha_{\mathrm{Baseline}}\left(\omega \right)$. Grey shaded regions indicate frequencies at which $\alpha_{\mathrm{Baseline}}\geq \alpha_{\mathrm{Dp}1}$, which are excluded from the curve.

**Figure A12:**
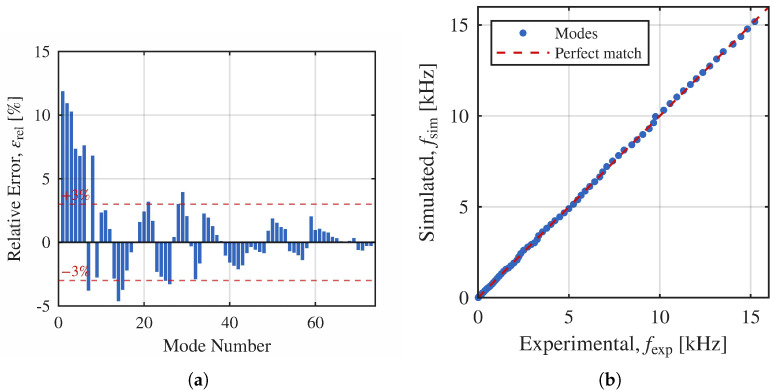
Comparison of simulated natural frequencies at $k_{\mathrm{opt}}=1.379\times 10^{6}$N/m against experimental measurements across the frequency range 6.81–15,300 Hz. (**a**) Per-mode relative frequency error $E^{\mathrm{rel}}\left(\%\right)$ between the simulated and experimental natural frequencies for all N=73 modes. (**b**) Parity plot comparing simulated and experimental natural frequencies; the dashed line denotes perfect agreement.

**Figure A13:**
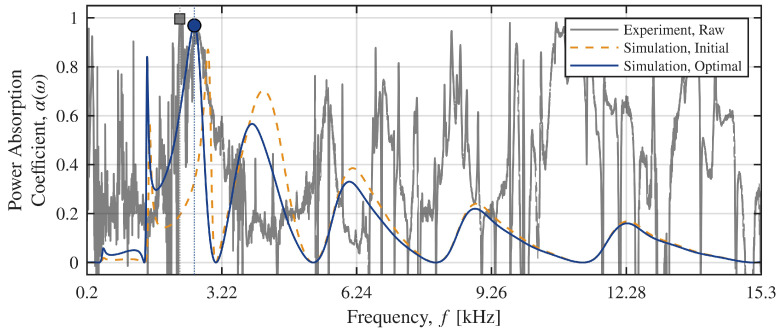
Comparison of the experimental (raw), initial-guess, and optimized power absorption coefficient α(ω). The initial simulation uses $k_{\mathrm{opt}}$ from Approach 1; markers indicate the first dominant peak for each curve.

**Figure A14:**
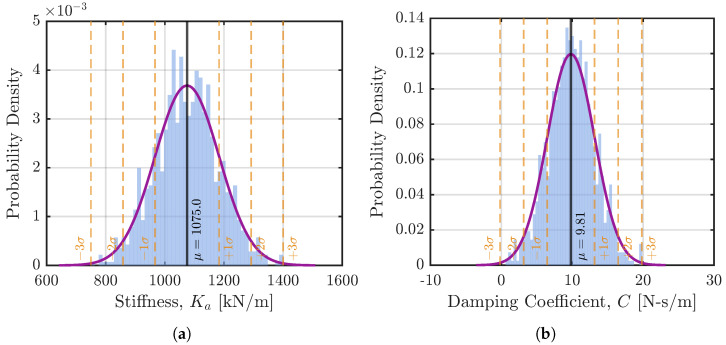
Normal distributions of the Monte Carlo samples (n=1080) for the stiffness and damping coefficient. Dashed lines indicate ±1σ, ±2σ, and ±3σ bounds. (**a**) Stiffness samples ($\mu_{k}=1075$ kN/m). (**b**) Damping coefficient samples ($\mu_{c}=9.815$ Ns/m).

**Figure A15:**
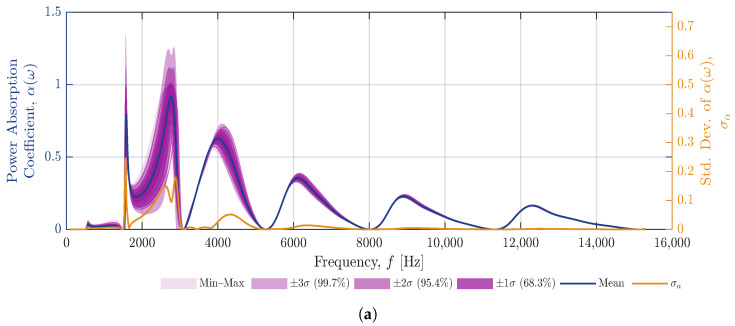

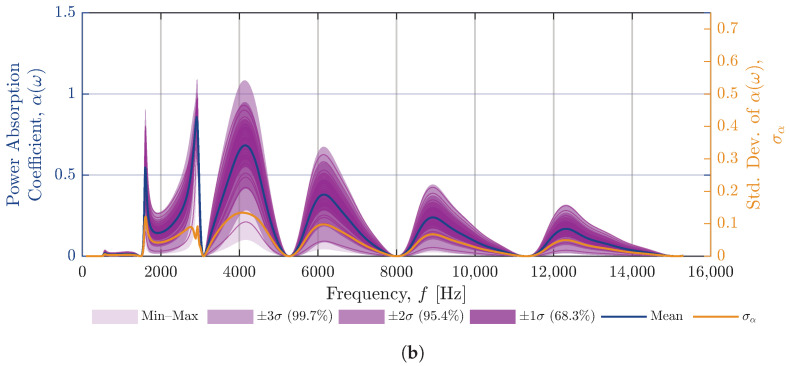
Ensemble of α(ω) realizations (n=1080) showing ±1σ, ±2σ, ±3σ confidence bands and standard deviation $\sigma_{\alpha }$ (right axis) for (**a**) stiffness variation and (**b**) damping variation.

## Data Availability

The raw data supporting the conclusions of this article will be made available by the authors upon request.

## Abbreviations

The following abbreviations are used in this manuscript:

DVA	Dynamic Vibration Absorber
FE	Finite Element
FFT	Fast Fourier Transform
FRF	Frequency Response Function
PD	Passive Damper
SLDV	Scanning Laser Doppler Vibrometer
FRVF	Fast Relaxed Vector Fitting
DMD	Dynamic Mode Decomposition
SINDy	Sparse Identification of Nonlinear Dynamics

## Appendix A. Glossary of Symbols

**Table A1:** Glossary of symbols.

Category	Symbol	Description
Geometric & Material Properties	L,W,H	Beam length, width, and thickness [m]
	E,G	Young’s modulus and Shear modulus [Pa]
	$\rho_{b}$	Material density [$kg/m^{3}$]
	ν	Poisson’s ratio [-]
	κ	Timoshenko shear coefficient [-]
	*A*	Cross-sectional area [$m^{2}$]
	*I*	Second moment of area [$m^{4}$]
Kinematics, Dynamics of Beam Model	w(x,t)	Transverse displacement [m]
	ψ(x,t)	Cross-section rotation [rad]
	$\dot{w},\;\dot{\psi }$	Time derivatives [m/s], [rad/s]
	M,C,K	Global mass, damping, and stiffness matrices
	$\alpha_{R},\;\beta_{R}$	Rayleigh damping coefficients in $C=\alpha_{R}M+\beta_{R}K$
	k,c	Dashpot stiffness and damping coefficients
	ω	Angular frequency [rad/s]
	*f*	Frequency [Hz]
Wave Decomposition Metrics	$k_{f}\left(\omega \right)$	Complex flexural wavenumber [1/m]
	λ(ω)	Flexural wavelength, λ=2π/Re{kf}
	Λ	Wave design matrix
	*n*	Number of sensors [-]
	$x_{i}$	Sensor location [m]
	$\hat{A},\;\hat{B}$	Forward and backward wave amplitudes
	r(ω)	Complex reflection coefficient [-]
	t(ω)	Complex transmission coefficient [-]
	ρ(ω)	Power reflection coefficient [-]
	τ(ω)	Power transmission coefficient [-]
	α(ω)	Power absorption coefficient [-]

## Appendix B. Model Validation

### Appendix B.1. Convergence Analysis

To validate the finite element (FE) representation of the host beam, the experimentally measured natural frequencies of the aluminum beam Section 2.1.2 were used as a reference. A mesh–refinement study was conducted by varying the FE discretization from 50 to 600 elements, with intermediate meshes of 100, 150, 200, 250, 300, 350, 400, 450, 500, 550, and 600 elements. For each mesh, the associated eigenvalues were computed and compared with those obtained from a 1000-element FE model, which served as the reference solution. As shown in Figure A1, the natural frequencies across the range of interest exhibit clear convergence as the mesh is refined, with the difference between the 400 and 450 element models being negligible.

Furthermore, Figure A2 demonstrates that the FRF predicted using the 400 element mesh aligns closely with the experimentally measured FRF at the corresponding location. On this basis, the host beam is modeled using 400 FE elements, and the resulting global mass and stiffness matrices are employed to simulate its dynamic response in MATLAB (v2025a).

### Appendix B.2. Experimental Error

A quantitative comparison of the experimental and simulated natural frequencies was performed to further assess the fidelity of the finite element (FE) model. The extracted frequencies and their corresponding relative errors are summarized in Table A2.

**Table A2:** Comparison of the experimentally measured and numerically simulated natural frequencies of the host beam.

Mode	Simulated Natural Frequency (Hz)	Experimental Natural Frequency (Hz)	$E^{\mathrm{rel}}\left(\%\right)$	$E_{L_{2}}^{\mathrm{rel}}\left(\%\right)$
1	6.6	5.9	12.5	12.5
2	18.1	16.1	12.6	12.5
3	35.4	31.7	11.6	11.8
4	58.3	53.2	9.5	10.3
5	86.6	81.5	6.2	8.0
6	120.6	114.3	5.5	6.8
7	160.5	152.8	5.0	6.0
8	206.8	198.7	4.0	5.2
9	259.5	247.1	5.0	5.1
10	318.5	305.7	4.2	4.8
11	383.5	368.2	4.2	4.6
12	454.0	439.5	3.3	4.2
13	529.0	513.7	3.0	3.8
14	607.9	591.3	2.8	3.6
15	691.0	675.3	2.3	3.3
16	780.2	764.6	2.0	3.0
17	877.1	856.4	2.4	2.9
18	982.0	957.0	2.6	2.8
19	1094.6	1064.5	2.8	2.8
20	1214.1	1179.2	3.0	2.8
21	1339.4	1304.2	2.7	2.8
22	1469.2	1431.6	2.6	2.8
23	1601.5	1561.5	2.6	2.7
24	1734.4	1692.9	2.5	2.7
25	1869.6	1819.3	2.8	2.7
26	2012.4	1980.0	1.6	2.5
27	2166.1	2122.0	2.1	2.5
28	2330.5	2295.0	1.5	2.3
29	2503.7	2464.4	1.6	2.2
30	2684.1	2637.7	1.8	2.2
31	2870.3	2812.5	2.1	2.2
32	3060.0	2988.3	2.4	2.2
33	3249.6	3175.8	2.3	2.2
34	3435.6	3382.3	1.6	2.1
35	3620.4	3577.6	1.2	2.0
36	3815.1	3789.1	0.7	1.9
37	4025.3	4003.4	0.5	1.8
38	4248.9	4210.0	0.9	1.7
39	4482.6	4429.2	1.2	1.7
40	4724.0	4624.0	2.2	1.7

For modes above the seventh, the percent relative error, $E^{\mathrm{rel}}\left(\%\right)$ ≤ 5%, indicates a strong agreement between the numerical and experimental results. The comparison of individual natural frequencies is shown in Figure A3a, while the associated $E^{\mathrm{rel}}\left(\%\right)$ and $E_{L_{2}}^{\mathrm{rel}}\left(\%\right)$ errors are presented in Figure A3b.

## Appendix C. Structural Damping Identification and Model Comparison

Accurate representation of structural damping is critical for reliable simulation of wave propagation in the host beam. Two damping models are considered in this work, namely proportional (Rayleigh) damping and modal damping, both parameterized using experimentally identified modal damping ratios $\zeta_{i}$. Rather than relying on conventional resonance identification techniques such as peak picking or circle fitting, the damping ratios are extracted using the Fast Relaxed Vector Fitting (FRVF) algorithm [62,63], which approximates the measured FRFs using a rational function

(A1) $$H\left(s\right)=\sum_{k=1}^{N_{p}}\frac{R_{k}}{s-a_{k}}+\frac{R_{k}^{*}}{s-a_{k}^{*}}+D+sE,$$

where s=jω, $R_{k}\in C$ are the residues, $a_{k}\in C$ are the poles, ${\left(\;\cdot \;\right)}^{*}$ denotes complex conjugation, and D,E∈R are terms that account for low and high frequency residual effects. From each converged complex conjugate pole pair $a_{k}=-\zeta_{k}\omega_{k}\pm j\omega_{k}\sqrt{1-\zeta_{k}^{2}}$, the undamped natural frequency $\omega_{k}=\left|a_{k}\right|$ and modal damping ratio ζk=−Re(ak)/|ak| are recovered directly. The resulting $\left(\omega_{i},\zeta_{i}\right)$ pairs serve as shared input for both damping models described in Appendix C.1 and Appendix C.2.

### Appendix C.1. Proportional Damping

Proportional damping is used for the host structure in our simulations. For an *n* degree of freedom system, the governing equation of motion is represented by Equation (2) where M, C, and K denote the mass, damping, and stiffness matrices, respectively, and x is the displacement vector. All matrices are of order N×N, with *N* being the number of measured modes. The damping matrix C is assumed to be proportional to M and K, represented by Equation (3) where $\alpha_{R}$ and $\beta_{R}$ are the proportional damping coefficients. Substituting Equation (3) into Equation (2) and following standard formulations [53,54,55] yields $2\zeta_{i}\omega_{i}=\alpha_{R}+\beta_{R}\omega_{i}^{2}$. Stacking the *N* modal relations gives Equation (A2),

(A2) $$\begin{array}{c} 2\left[\begin{array}{c} \zeta_{1}\omega_{1}\\ \zeta_{2}\omega_{2}\\ \zeta_{3}\omega_{3}\\ \vdots \\ \zeta_{N}\omega_{N} \end{array}\right]=\left[\begin{array}{cc} 1 & \omega_{1}^{2}\\ 1 & \omega_{2}^{2}\\ 1 & \omega_{3}^{2}\\ \vdots & \vdots \\ 1 & \omega_{N}^{2} \end{array}\right]\left\{\begin{array}{c} \alpha_{R}\\ \beta_{R} \end{array}\right\}. \end{array}$$

Solving the least squares system using the $\zeta_{i}$ values extracted by FRVF yields

(A3) $$\begin{array}{c} \alpha_{R}=0.569\;1/s,\;\beta_{R}=7.846\times 10^{-8}\;s. \end{array}$$

The damping coefficients from the experiment and the simulation are compared in Figure A4. The contributions to $\zeta_{i}$ from the mass and stiffness matrices follow

$$\begin{array}{c} \zeta_{m}=\frac{\alpha_{R}}{{2\omega }_{i}},\;\zeta_{k}=\frac{\beta_{R}\omega_{i}}{2}, \end{array}$$

with $\zeta_{m}$ and $\zeta_{k}$ denoting the respective effects; their simulated trends are also shown in Figure A4.

### Appendix C.2. Modal Damping

The proportional damping assumption enforces a smooth variation of $\zeta_{i}$ with frequency, governed entirely by $\alpha_{R}$ and $\beta_{R}$, and therefore cannot capture the individual damping variation from mode to mode arising from material inhomogeneity or localized dissipation mechanisms. The modal damping model relaxes this constraint by assigning each mode its own experimentally measured $\zeta_{i}$, thereby accommodating damping behavior that is not proportional without introducing additional free parameters. Let $\Phi =[\phi_{1},\phi_{2},\ldots ,\phi_{N}]$ be the mass normalized mode shapes satisfying

(A4) $$\Phi^{⊤}M\Phi =I,\;\Phi^{⊤}K\Phi =\mathrm{diag}\left(\omega_{i}^{2}\right),$$

where I is the N×N identity matrix. The modal damping matrix $C_{m}$ is assembled via the spectral expansion

(A5) $$C_{m}=M\Phi \;\mathrm{diag}\left(2\zeta_{i}\omega_{i}\right)\;\Phi^{⊤}M,$$

which, by construction, satisfies $\Phi^{⊤}C_{m}\Phi =\mathrm{diag}\left(2\zeta_{i}\omega_{i}\right)$, recovering the measured damping ratios exactly in modal coordinates. The $\zeta_{i}$ values entering Equation (A5) are those extracted by FRVF as described at the opening of this section. Unlike the Rayleigh model with its two scalar parameters, $C_{m}$ captures the full variation in damping from mode to mode as observed experimentally in Figure A4, and is therefore expected to provide a more faithful representation of the beam dissipation characteristics across the 0–15.3 kHz frequency range.

### Appendix C.3. Effect of Damping Models on Wave Propagation Characteristics

The influence of both damping models on the host beam response is assessed by comparing FRFs from three FE configurations, namely the undamped, proportionally damped Equations (3) and (A3), and modal damped Equation (A5) models, as shown in Figure A5. These responses form the basis for computing the reflection, power reflection, and power absorption coefficients of the host beam, following the procedure outlined in Section 2.2.3.

For a conservative system, the power reflection coefficient ρ(ω) is expected to be unity at all frequencies, as confirmed by the undamped model in Figure A6d. Structural damping reduces ρ(ω) progressively below unity at higher frequencies due to energy dissipation; this behavior is evident for both the proportionally damped and modal damped models in Figure A6e,f. Correspondingly, the power absorption coefficient α(ω) remains zero for the undamped model Figure A6g, while it increases from zero at higher frequencies for both damped configurations Figure A6h,i. The undamped FE model is therefore adopted as the baseline for subsequent analysis, representing the ideal conservative condition against which the performance of the passive damper (PD) is evaluated.

## Appendix D. Sensor Layout and Wave Decomposition Reliability

The reliability of the estimated wave coefficients depends critically on both the spatial arrangement and the number of sensors employed in the wave decomposition. This appendix examines two complementary aspects of this dependence, namely, the numerical conditioning of the design matrix Λ as a function of sensor spacing and the sensitivity of the power absorption coefficient α(ω) to the choice of sensor subset across all 9n combinations for n∈{2,3,…,8}. Together, these analysis justify the sensor configuration adopted in the main body of this work, comprising the use of non-uniform spacing to maintain a well-conditioned design matrix across the frequency range of interest and the selection of all nine available sensors to minimize estimation uncertainty in α(ω).

### Appendix D.1. Condition Number Evaluation for Wave Decomposition

From Equation (12) in Section 2.2.3, evaluating the reflection coefficient for the beam with an end-mounted dashpot reduces to solving a linear system of the form Λx=b. The reliability of this solution depends on the conditioning of Λ and, in particular, on the behavior of its pseudoinverse $\Lambda^{†}$. The condition number κ(Λ) quantifies the sensitivity of the solution to perturbations in either Λ or *b*, and therefore provides a direct indication of the numerical stability of the extracted wave coefficients. As a rule of thumb, if $\kappa \left(\Lambda \right)\approx 10^{m}$, up to *m* digits of accuracy may be lost in the computed solution. Because Λ depends on the sensor layout and the underlying wavenumber, different sensor layouts can be employed to maintain a low and stable condition number.

Figure A7 compares the condition number for the uniformly spaced sensor layout in Figure A7a with that for the non-uniform layout in Figure A7b. The uniformly spaced arrangement yields a large condition number across much of the frequency range, indicating poor numerical conditioning and reduced reliability in the wave decomposition results. By contrast, the non-uniform arrangement maintains a substantially lower condition number and therefore provides a more stable basis for wave coefficient estimation. For this reason, the non-uniform sensor layout is adopted, subject to the placement constraints described in Section 2.2.2, to ensure stable and reliable estimation of the wave coefficients.

### Appendix D.2. Effect of Sensor Count on Power Absorption Coefficient Uncertainty

The wave decomposition of Section 2.2.3 requires at least two sensors to resolve the forward and backward propagating wave amplitudes, and the accuracy of the estimated absorption coefficient $\alpha \left(\omega \right)=1-{\left|r\left(\omega \right)\right|}^{2}$ improves with the sensor count *n*, since a larger *n* yields a more overdetermined least squares system that is less sensitive to measurement noise and sensor placement.

To quantify this dependence, all 9n subsets of the nine sensor locations in Table A3 were evaluated independently for each n∈{3,4,…,8}, and the resulting ensemble of α(ω) estimates yielded the mean $\bar{\alpha }\left(\omega \right)$, the standard deviation $\sigma_{\alpha }\left(\omega \right)$, the symmetric intervals $\bar{\alpha }\pm k\sigma_{\alpha }$ for k∈{1,2,3} (coverage probabilities of 68.3%, 95.4%, and 99.7%), and the minimum to maximum envelope.

**Table A3:** Far-field sensor locations used for wave decomposition. Distances are measured from the left-clamped end of the beam.

	Sensor Location, $x_{i}$
1	0.350 m
2	0.361 m
3	0.372 m
4	0.383 m
5	0.395 m
6	0.407 m
7	0.419 m
8	0.432 m
9	0.448 m

For n=2 the system is exactly determined, and the spread is large, with $\sigma_{\alpha }$ reaching 0.1 and the envelope spanning nearly the full admissible interval [0,1], confirming that two sensors are insufficient. Figure A8 presents n=3 through n=8. The first overdetermination at n=3 reduces the uncertainty markedly, though $\sigma_{\alpha }$ still spikes near the structural resonances where the condition number of Λ is elevated (Appendix D.1); the bands then tighten progressively from n=4 to 6 and become indistinguishable from the mean for n≥7, where $\sigma_{\alpha }$ stays below about 0.005. These results establish a monotonic reduction in estimation uncertainty with sensor count and show that n≥5 sensors provide a robust, stable estimate of α(ω) for the configuration studied.

## Appendix E. Post-Processing and Filtering of Experimental Power Absorption Coefficients

Applying the wave decomposition directly to experimental FRFs frequently produces values of the power absorption coefficient α(ω) outside the physically admissible range of [0,1]. These nonphysical values arise primarily near structural resonances and anti-resonances, where rapid amplitude variations in the measured FRF cause small measurement errors to propagate disproportionately into the wave amplitude ratio that defines the reflection coefficient. A post-processing procedure is therefore applied to the raw wave decomposition coefficients to retain only physically meaningful and numerically reliable frequencies.

The ordinary coherence function serves as a frequency-resolved indicator of signal quality. The coherence is computed at each sensor location and averaged spatially to produce a mean coherence spectrum ${\bar{\gamma }}^{2}\left(f\right)$, shown in Figure A10. Frequencies at which ${\bar{\gamma }}^{2}\leq 0.90$ in either the dashpot or the baseline dataset are excluded from all subsequent processing, ensuring that comparisons between the two configurations are drawn from a common set of reliable frequencies.

Before smoothing, frequencies at which α(ω) lie outside the broad interval [−5,5] are removed to prevent large-amplitude outliers from contaminating adjacent bins through the convolution window of the subsequent filter. These extreme values arise near structural anti-resonances, where the FRF amplitude approaches a local minimum, and the least-squares wave decomposition becomes poorly conditioned. The pre-screened wave coefficients are then smoothed using a Savitzky–Golay filter with polynomial order 3 and a frame length of 151 bins, applied independently to the real and imaginary parts of the complex reflection coefficient and to the real-valued power coefficients. This filter preserves genuine broadband trends while attenuating narrowband noise scatter and is therefore preferred over a simple moving average. The combined effect of these two steps is illustrated in Figure A9.

Following coherence filtering and smoothing, the vast majority of frequencies yield absorption coefficients within the physical range [0,1]. A final validity window of α(ω)∈[−0.05,1.05] is applied to exclude any residual outliers, with the same criterion applied independently to ρ(ω). The classified dataset and the frequency-resolved validity distribution are shown in Figure A10.

To isolate the energy dissipation contribution of the dashpot from that of the host beam, the net power absorption is defined as $\Delta \alpha \left(\omega \right)=\alpha_{\mathrm{Dp}1}\left(\omega \right)-\alpha_{\mathrm{Baseline}}\left(\omega \right)$. Only frequencies at which the dashpot configuration exhibits strictly higher absorption than the baseline are retained; frequencies at which the baseline absorption equals or exceeds that of the dashpot are shaded and excluded, as they do not represent a positive contribution from the dashpot. The resulting net absorption spectrum is shown in Figure A11, and it reveals the frequency bands over which the dashpot provides measurable additional wave energy dissipation relative to the host beam alone.

## Appendix F. Determination of Spring Damper Parameters

The air damper (dashpot) employed in this study introduces both stiffness and damping contributions at the attachment point on the host beam. Direct mechanical calibration of these parameters proved experimentally impractical; the manufacturer’s datasheet [64] provided only a range for the damping coefficient without specifying the stiffness of the dashpot. Two independent identification approaches were therefore pursued, each exploiting a distinct observable quantity. The first approach determines the attachment stiffness *K* from shifts in the beam’s measured natural frequencies; the second approach determines both *K* and the viscous damping coefficient *C* jointly from the experimentally measured power absorption coefficient α(ω). These two approaches are complementary; Approach 1 provides a high-confidence estimate of *K* from a large frequency dataset, while Approach 2 supplies *C*, which cannot be inferred from undamped natural frequencies.

### Appendix F.1. Approach 1: Natural Frequency Matching (Stiffness Identification)

The undamped natural frequencies of a beam are sensitive to any attached stiffness but remain largely insensitive to viscous damping for the moderate damping ratios encountered in the present configuration. This physical decoupling allows the PD stiffness *k* to be identified independently from its damping coefficient *c*. Frequency response measurements of the beam with the dashpot attached yielded N=73 experimental natural frequencies $f_{i}^{\;\exp}$, i=1,…,N, spanning 6.81–15,300 Hz. The Timoshenko beam finite element model was augmented with a passive damper (PD) of stiffness *k* at the dashpot attachment degree of freedom. The modified stiffness matrix takes the form

(A6) $$K\left(j\right)=K_{0}+k_{j}\;B_{pd},$$

where $K_{0}$ is the baseline assembled stiffness matrix and $B_{pd}$ is the Boolean coupling matrix that localizes the spring contribution to the attachment degree of freedom [56]. The corresponding simulated natural frequencies $f_{i}^{\;\mathrm{sim}}\left(k_{j}\right)$ are obtained from the undamped generalized eigenvalue problem

(A7) $$K\left(j\right)\;\phi =\omega^{2}\;M\;\phi ,$$

where M is the global mass matrix, ϕ is the modeshape vector, and $\omega_{i}=2\pi f_{i}^{\;\mathrm{sim}}$ is the angular natural frequency of the *i*-th mode. The optimal stiffness $k_{\mathrm{opt}}$ is found by minimizing the relative frequency error over all *N* identified modes,

(A8) $$k_{\mathrm{opt}}=\min_{k_{j}\;\in \;R^{+}}\;J\left(k_{j}\right)\;J\left(k_{j}\right)=\sqrt{\;\sum_{i=1}^{N}{\left(\frac{f_{i}^{\;\mathrm{sim}}\left(k_{j}\right)-f_{i}^{\;\exp}}{f_{i}^{\;\exp}}\right)}^{\;2}\;},$$

where $J(k_{j})$ is the objective function evaluated at the *j*-th candidate stiffness value, $j=1,\ldots ,N_{\mathrm{iter}}$. Simulated and experimental frequencies were matched using a greedy nearest-frequency assignment; each experimental frequency was paired with the closest available simulated frequency in the relative-error sense, without replacement, making the objective robust to mode crossings as *k* varies.

The identified optimal stiffness was found to be $k_{\mathrm{opt}}=1.379\times 10^{6}$ N/m. The per-mode relative frequency error $\varepsilon_{i}$ is shown in Figure A12a, and the corresponding parity plot comparing $f_{i}^{\;\mathrm{sim}}\left(k_{\mathrm{opt}}\right)$ against $f_{i}^{\;\exp}$ for all N=73 modes is shown in Figure A12b. The simulated natural frequencies show close agreement with the experimental values across the full frequency range, with the majority of modes falling well within the ±3% error band. The comparatively higher errors observed at the lower modes can be attributed to boundary condition uncertainties inherent in the experimental setup, where small deviations from ideal support conditions have a proportionally greater influence on the lower resonances than on the higher ones.

### Appendix F.2. Approach 2: Power Absorption Coefficient Matching (Stiffness & Damping Identification)

The power absorption coefficient α(ω) is sensitive to both the dashpot stiffness *k* and the viscous damping coefficient *c*, making it suitable for their simultaneous identification. The experimental α(ω) curve exhibits a dominant first peak, at which the dashpot absorbs the greatest proportion of the incident wave power; this peak was therefore considered the most physically informative feature for parameter identification. The optimal parameter pair $\left(k_{\mathrm{opt}},\;c_{\mathrm{opt}}\right)$ is found by minimizing the simulated and experimental first peak,

(A9) $$\left(k_{\mathrm{opt}},\;c_{\mathrm{opt}}\right)=\min_{k,\;c\;\in \;R^{+}}\;F\left(k,c\right),\;F\left(k,c\right)=w_{f}\;E_{f}^{2}+w_{\alpha }\;E_{\alpha }^{2},$$

where $E_{f}$ and $E_{\alpha }$ are the relative frequency error and absolute amplitude error at the first dominant peak,

(A10) $$E_{f}=\frac{f_{\mathrm{peak}}^{\;\mathrm{sim}}-f_{\mathrm{peak}}^{\;\exp}}{f_{\mathrm{peak}}^{\;\exp}},\;E_{\alpha }=\alpha_{\mathrm{peak}}^{\;\mathrm{sim}}-\alpha_{\mathrm{peak}}^{\;\exp},$$

and $w_{f}$ and $w_{\alpha }$ are scalar weights. The search bounds on *k* were centered around $k_{\mathrm{opt}}$ from Approach 1, while the bounds on *c* were prescribed by the manufacturer’s datasheet range [64]. A multi-start gradient-based solver was employed over k∈[500,30000] kN/m and c∈[0,2000] Ns/m to obtain $\left(k_{\mathrm{opt}},\;c_{\mathrm{opt}}\right)$. The initial simulation used *k* identified from Approach 1 as the starting guess, with the experimental, initial, and optimized α(ω) curves compared in Figure A13.

The identified parameters are $k_{\mathrm{opt}}=821.07$ kN/m and $c_{\mathrm{opt}}=9.8149$ Ns/m. The optimized simulation closely reproduces the frequency and amplitude of the first dominant absorption peak, as seen in Figure A13. The identified parameters from both approaches are summarized in Table A4.

**Table A4:** Summary of identified spring–damper parameters from Approaches 1 and 2.

	Approach 1	Approach 2
Stiffness, $k_{\mathrm{opt}}$ (kN/m)	1379.01	821.07
Damping Coefficient, $c_{\mathrm{opt}}$ (Ns/m)	—	9.8149

The close agreement between the stiffness estimates from both approaches provides mutual validation of the identification procedures. Some uncertainty in $k_{\mathrm{opt}}$ is nonetheless expected, as Approach 1 is sensitive to boundary condition uncertainties in the lower modes (Figure A12a), while Approach 2 is sensitive to the accuracy of the wave decomposition model near the first absorption peak. The damping coefficient $c_{\mathrm{opt}}$ carries additional uncertainty arising from the limited resolution of the manufacturer’s datasheet range, which defines the search bounds but does not uniquely constrain *c*. The stiffness $k_{\mathrm{opt}}$ from Approach 1 and the damping coefficient $c_{\mathrm{opt}}$ from Approach 2 were adopted in all simulations presented in the main body of this paper.

## Appendix G. Parametric Uncertainty Analysis of the Spring–Damper

The parameters identified in Appendix F carry inherent uncertainty; the stiffness $k_{\mathrm{opt}}$ is sensitive to boundary condition assumptions, and the damping coefficient $c_{\mathrm{opt}}$ is constrained only by the manufacturer’s datasheet range. To assess the influence of this uncertainty on the power absorption coefficient α(ω), a Monte-Carlo analysis was performed for each parameter independently, with n=1080 samples. In each analysis, one parameter was varied while the other was held fixed at its identified value.

The stiffness was modeled as $k∼N\mu_{k},\;\sigma_{k}^{2})$ with bounds $k_{\min}=750$ kN/m and $k_{\max}=1400$ kN/m spanning the range of estimates from both approaches, giving $\mu_{k}=1075$ kN/m and $\sigma_{k}=\left(k_{\max}-\mu_{k}\right)/3$ such that the ±3σ interval covers the full bound range. The damping coefficient was modeled as $c∼N(\mu_{c},\;\sigma_{c}^{2})$ with $\mu_{c}=9.815$ Ns/m and $\sigma_{c}=10/3$ Ns/m, so that the ±3σ interval spans [0,20] Ns/m with the values specified within the manufacturer’s datasheet [64]. In both cases, samples were clipped to [μ−3σ,μ+3σ]. The resulting distributions are shown in Figure A14, and the corresponding α(ω) ensembles are shown in Figure A15.

As shown in Figure A15a,b, stiffness uncertainty primarily shifts the frequency location of the absorption peaks, whereas damping uncertainty primarily affects their amplitude. In both cases, the ±1σ band remains narrow near the first dominant peak and $\sigma_{\alpha }$ is concentrated at the peak frequencies, approaching zero in the troughs. These results confirm that α(ω) is robust to the level of parametric uncertainty inherent in the identification, and that the adopted values $\left(k_{\mathrm{opt}},\;c_{\mathrm{opt}}\right)$ provide a representative characterization of the dashpot.
